# GSDMD deficiency attenuates BPD by suppressing macrophage pyroptosis and promoting M2 polarization

**DOI:** 10.1038/s41420-025-02872-4

**Published:** 2025-12-04

**Authors:** Xinyi Yang, Xinru Wang, Yihang Yang, Yue Ma, Xinli Liu, Dandan Mo, Chunbao Guo, Wenli Han

**Affiliations:** 1 School of Pharmacy, Chongqing Medical University, Chongqing, PR China; 2 Key Laboratory for Biochemistry and Molecular, College of Pharmacy, Chongqing Medical University, Chongqing, PR China; 3 Department of Pediatrics, Chongqing Health Center for Women and Children, Chongqing, PR China; 4 School of Pharmacy and Bioengineering, Chongqing University of Technology, Chongqing, PR China; 5 Department of Pediatric Surgery, The Affiliated Hospital of Qingdao University, Qingdao, PR China

**Keywords:** Chronic inflammation, Respiratory tract diseases

## Abstract

Bronchopulmonary dysplasia (BPD), a frequent complication in preterm infants receiving supplemental oxygen, is characterized by hyper-activation of macrophage inflammasomes, exuberant release of pro-inflammatory cytokines such as interleukin-1β (IL-1β), and Gasdermin D (GSDMD)-driven pyroptosis. However, the precise contribution of macrophage pyroptosis to BPD pathogenesis remains incompletely defined, and effective pharmacological interventions are still lacking. Using neonatal C57BL/6 wild-type (WT) and GSDMD-knockout (GSDMD^−/−^) mice, we established a hyperoxia-induced BPD model (85% FiO₂, 14 days) and administered the GSDMD inhibitor disulfiram (50 mg kg⁻¹ intraperitoneally, once daily for 7 days). In vivo, we assessed lung histopathology, IL-1β levels, alveolarization, and vascular development; ex vivo, we isolated bone-marrow-derived macrophages (BMDMs) to quantify pyroptotic markers, M1/M2 polarization, and antibacterial capacity. GSDMD deletion or disulfiram treatment significantly attenuated macrophage and neutrophil infiltration, decreased pulmonary IL-1β concentrations, improved alveolar architecture and vascular density, and reduced overall cell death. BMDMs from GSDMD^−/−^ mice displayed diminished M1 polarization, enhanced bacterial killing, yet unaltered zymosan phagocytosis. Collectively, these findings identify GSDMD-mediated macrophage pyroptosis as a critical driver of BPD-related lung injury. Targeted GSDMD inhibition, whether genetic or pharmacologic, alleviates experimental BPD by down-regulating IL-1β and promoting alveolar development, thereby providing a promising therapeutic avenue for this devastating neonatal disorder.

## Introduction

Bronchopulmonary dysplasia (BPD) is a lung disease that mainly affects premature infants, defined as needing supplemental oxygen beyond 28 days after birth or at 36 weeks’ postmenstrual age [1]. Owing to limited research, progress in prevention and treatment remains slow [2]. Clarifying its mechanisms and identifying new therapeutic targets are therefore vital for improving clinical outcomes. In severe cases of BPD, as well as those leading to fatal outcomes, inflammatory cascades and heightened levels of inflammatory cytokines, such as IL-6 and TNF-α, are commonly observed [3]. Apart from infections such as intrauterine inflammation or postnatal sepsis, lung development can also be compromised by sterile inflammation triggered by hyperoxia. Suppressing hyperoxic inflammation is critical for protecting the lungs of oxygen-dependent preterm infants. Immunomodulation, macrolides, and cellular therapies have shown promise in BPD [4], yet the absence of effective immune targets hampers drug development.

Pyroptosis is a form of programmed cell death characterized by the release of the pro-inflammatory cytokine IL-1β, which exacerbates tissue inflammation [5]. Elevated IL-1β levels correlate with the severity of BPD [6]. Additionally, IL-6 and CXCL10 signaling pathways are involved in macrophage polarization, alveolar damage, and fibrotic remodeling, making them promising therapeutic targets [7, 8]. The NLRP3 inflammasome and Caspases are pivotal in the process of pyroptosis, wherein activated Caspases trigger the cleavage of GSDMD [9]. This cleavage results in the formation of pores in cell membranes, facilitating the release of pro-inflammatory cytokines such as IL-1β and IL-18 [10–12]. Mice deficient in inflammasome constituents, including NLRP3 or Caspase-1/11, exhibit decreased susceptibility to endotoxemia and sepsis [13, 14], whereas GSDMD-driven pyroptosis propagates inflammation and tissue damage in acute lung injury models [15]. Targeting GSDMD, therefore, represents a promising therapeutic avenue for pulmonary inflammatory disorders. Moreover, Caspase-1 inhibitors can block GSDMD-mediated pyroptosis and attenuate hyperoxia-induced lung and brain injury, offering a potential approach for managing oxygen-related complications in preterm infants [16, 17].

Our previous study highlighted the exaggerated inflammatory reaction of macrophages in premature infants as a critical element in the development of BPD [18, 19]. In an inflammatory milieu, macrophages polarize toward the M1 phenotype and secrete abundant cytokines via GSDMD pores, thereby exacerbating tissue injury [20, 21]. Nevertheless, the precise roles of GSDMD-mediated pyroptosis and its interplay with M1 polarization in the pathogenesis of BPD remain incompletely defined. Recent evidence implicates GSDMD-mediated pyroptosis in neonatal lung injury [22]. We hypothesize that GSDMD drives BPD progression by simultaneously inducing macrophage pyroptosis and promoting M1 polarization.

This study evaluates GSDMD’s impact on pulmonary injury and macrophage function, focusing on macrophage infiltration, pyroptosis, and crosstalk with pro-inflammatory cytokines. Targeting GSDMD may thus represent a novel therapeutic strategy for neonatal BPD.

## Results

### GSDMD knockout protects against BPD

**Fig. 1:**
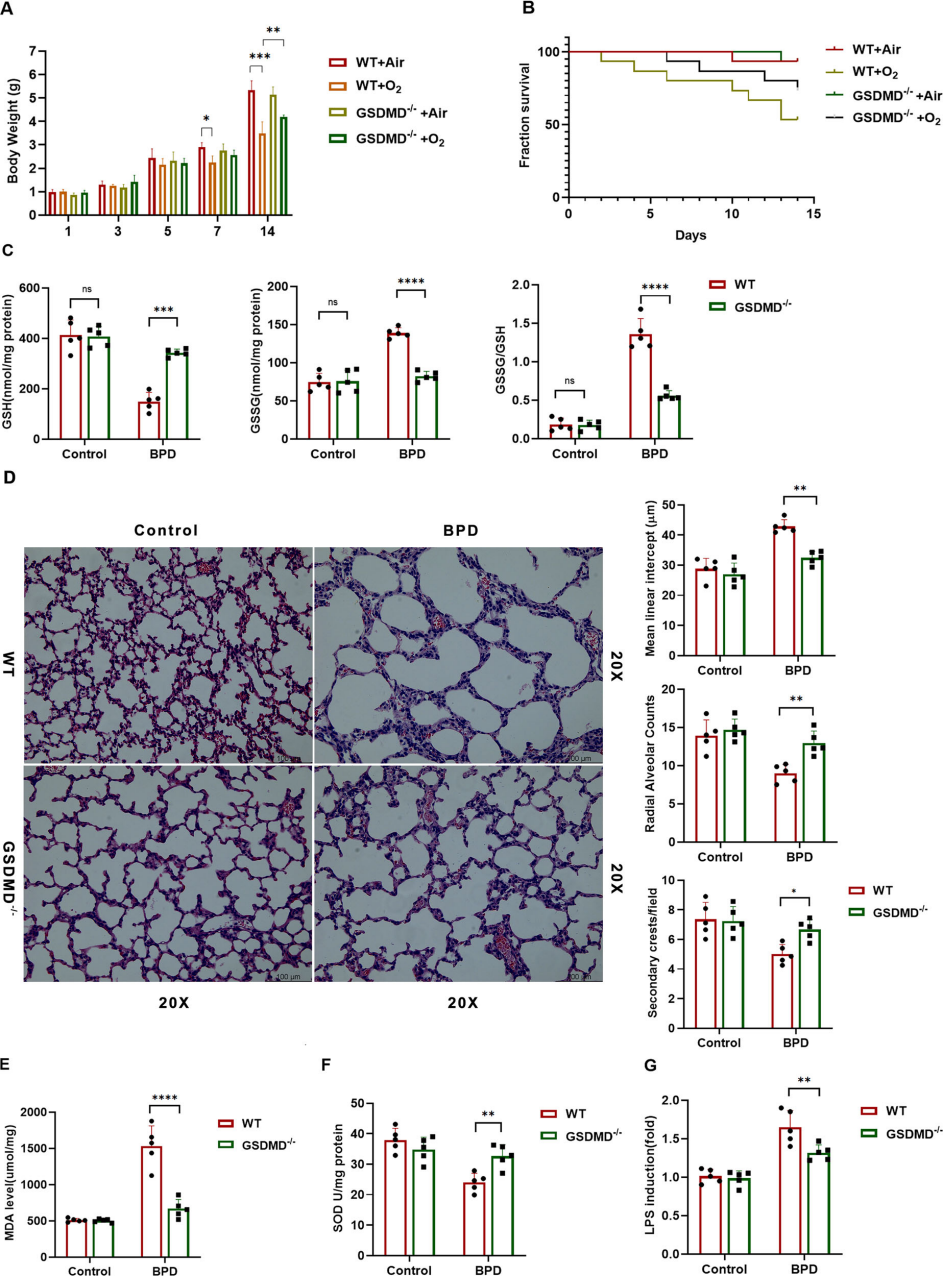
GSDMD knockout protects against BPD. **A** The time-course of pup body weight across treatment groups is depicted. Columns represent the mean ± SD of multiple independent experiments (*n* = 9–10 pups per group). *P < 0.05, **P < 0.01, ***P < 0.001, one-way ANOVA. **B** Kaplan–Meier survival curves were generated for pups with experimental BPD. Data are expressed as the percentage survival of the total cohort (*n* = 15 pups per group, log-rank [Mantel–Cox] test). **C** GSH, GSSG, and the GSSG/GSH ratio (nmol/mg of protein) were quantified in lung homogenates as detailed in the Methods. Columns show the mean ± SD of multiple independent experiments (*n* = 5 pups per group). ***P < 0.001, ****P < 0.0001, one-way ANOVA. **D** Representative H&E-stained lung sections are shown for each treatment group (*n* = 5 animals, 10 fields per animal; magnification = 20×; scale bar = 100 μm). Alveolar development was evaluated morphometrically (*n* = 5 animals, 10 fields per animal) by determining MLI, RAC and secondary crest counts per field. Columns represent the mean ± SD of multiple independent experiments. *P < 0.05, **P < 0.01, one-way ANOVA. **E** MDA levels and **F**. SOD activities were measured in whole-lung homogenates as described. Columns depict the mean ± SD of multiple independent experiments (*n* = 5 pups per group). **P < 0.01, ****P < 0.0001, one-way ANOVA. **G** Endotoxin (LPS) levels in the indicated groups were quantified as described. Columns show the mean ± SD of multiple independent experiments (*n* = 5 pups per group). **P < 0.01, one-way ANOVA.

As illustrated in Fig. 1A, neonatal mice exposed to hyperoxia exhibited pronounced signs of illness and notable weight reduction. In contrast, pups lacking GSDMD showed enhanced overall physical development. Survival analysis indicated a significantly higher survival rate in GSDMD^−/−^ mice after the onset of BPD compared to their WT counterparts (Fig. 1B). Oxidative stress plays a pivotal role in the pathogenesis of BPD. Initially, we evaluated the levels of the tripeptide GSH and its oxidized form (GSSG) in the experimental BPD model. As depicted in Fig. 1C, GSH levels were substantially reduced under hyperoxia exposure, while GSSG levels increased in mice exposed to oxygen compared to those exposed to air. Moreover, the GSSG/GSH ratio rose, indicating heightened oxidative stress during hyperoxia exposure (Fig. 1C). Interestingly, GSDMD knockout further decreased GSH levels while increasing GSSG content and the GSSG/GSH ratio (Fig. 1C). This may reflect compensatory redox shifts due to reduced pyroptotic ROS bursts.

Histopathological examination revealed alveolar simplification and dysalveolarized patterns in neonatal mice exposed to hyperoxia for 21 days. Notably, GSDMD^−/−^ mice exhibited a marked reduction in alveolar simplification, as assessed by the secondary crest/field ratio, making them nearly indistinguishable from control animals (Fig. 1D). Furthermore, morphological analysis indicated that GSDMD knockout increased the number of alveoli (RACs) and decreased alveolar size (MLI) (Fig. 1D). Consistent with these observations, elevated levels of MDA, a lipid peroxidation marker, and increased protein carbonyl content, indicative of protein oxidative damage, were detected in lung tissues of mice with experimental BPD. These effects were significantly attenuated by GSDMD knockout (Fig. 1E). Moreover, SOD activity was significantly enhanced in GSDMD^−/−^ mice (Fig. 1F), highlighting its protective role in the development of BPD. GSDMD knockout also led to reduced levels of the bacterial translocation marker LPS (Fig. 1G), emphasizing the critical involvement of GSDMD-mediated signaling in lung health.

### GSDMD knockout attenuates pulmonary macrophage mobilization and infiltration during BPD development

**Fig. 2:**
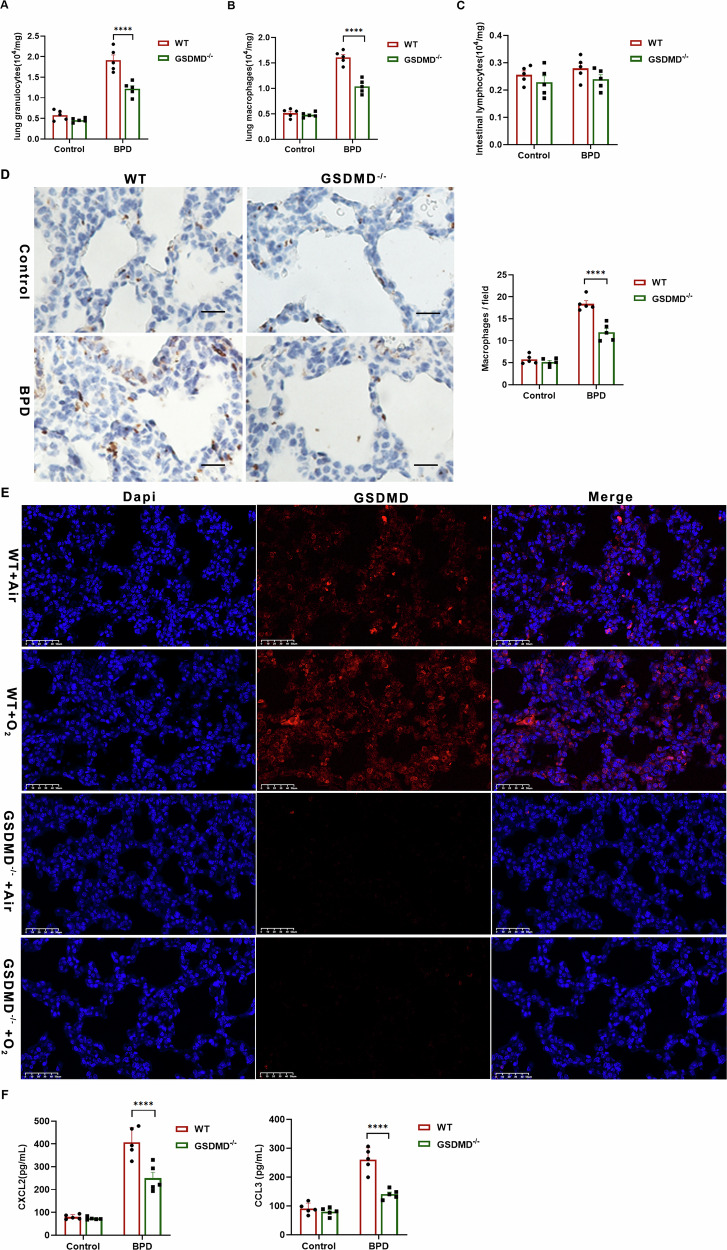
GSDMD knockout attenuates pulmonary macrophage mobilization and infiltration during BPD development. **A** Granulocyte counts in pups from the indicated treatment groups were quantified. **B** Macrophage numbers were determined in the same samples. **C** Lymphocyte levels were measured for comparison. Data are presented as mean ± SD of multiple independent experiments; ****P < 0.0001 (one-way ANOVA). **D** Representative F4/80 immunostaining identifying macrophages in lung sections from pups in the indicated groups. For each sample, 3–4 random fields were captured at 20× magnification; scale bar = 50 µm. The percentage of F4/80⁺ macrophages was quantified (*n* = 5 per group). Columns show mean ± SD of multiple independent experiments; ****p < 0.0001 (one-way ANOVA). **E** Immunofluorescence co-localization images of GSDMD (red) and DAPI (blue, nuclear stain) in lung tissue. Images were acquired at 20× magnification; scale bar = 50 µm. **F** Concentrations of chemokines CXCL2 and CCL3 in lung homogenates from mice in the indicated groups (*n* = 5 per group). Data are expressed as mean ± SD of multiple independent experiments; ****P < 0.0001 (one-way ANOVA).

Prior research has confirmed a notable presence of macrophage infiltration in BPD. To investigate the potential impact of GSDMD knockout on inflammatory cell recruitment, we examined pulmonary specimens for granulocytes (identified by anti-Ly6G monoclonal antibody), macrophages (identified by anti-F4/80 monoclonal antibody), and lymphocytes (CD3 positive). Our analysis revealed that BPD was linked to marked macrophage infiltration and a slight elevation in granulocyte levels in the lung, as depicted in Fig. 2A, B. Nonetheless, there was no significant disparity in lymphocyte counts between BPD and control offspring (Fig. 2C). Notably, GSDMD^−/−^ mice displayed diminished macrophage populations compared to WT pups (Fig. 2B). Examination of lung tissue sections further demonstrated substantial macrophage infiltration in WT pups exposed to hyperoxia in contrast to other cohorts, In GSDMD^−/−^mice, the macrophage count was lower than that in other groups (Fig. 2D). Immunofluorescence analysis further confirmed the reduced macrophage infiltration in GSDMD^−/−^ mice (Fig. 2E). These results indicate that the knockout of GSDMD hinders the migration of inflammatory cells, specifically macrophages, into the pulmonary tissue. GSDMD is recognized for its role in mediating the secretion of chemokines and cytokines that facilitate the recruitment of macrophages and neutrophils. Our investigation involved assessing the concentrations of diverse cytokines and chemokines in both peripheral blood and lung specimens. BPD markedly increased the expression of the monocyte-attracting chemokines CXCL2 and CCL3 (Fig. 2F). Importantly, the heightened levels of these chemokines, particularly CXCL2 and CCL3, were substantially diminished in GSDMD^−/−^ mice, underscoring the pivotal involvement of GSDMD in regulating the inflammatory reaction (Fig. 2F).

### GSDMD knockout alters macrophage phenotype and activation

**Fig. 3:**
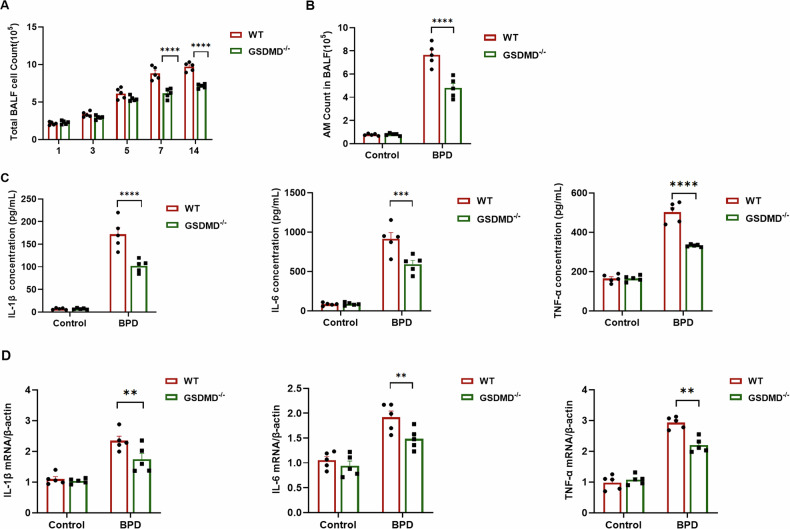
GSDMD knockout alters macrophage phenotype and activation. **A** BALF was collected from WT and GSDMD⁻^/^⁻ mice at the indicated time points, and total cell counts were obtained with an automated cytometer. Columns represent the mean ± SD of multiple independent experiments (*n* = 5 mice per group); ****P < 0.0001, one-way ANOVA. **B** AMs in BALF were further enumerated and are presented as mean ± SD; ****P < 0.0001, one-way ANOVA. **C** Levels of IL-1β, IL-6, and TNF-α in cell-free BALF were measured by ELISA. Columns depict the mean ± SD of multiple independent experiments (*n* = 5 mice per group); ***P < 0.001, ****P < 0.0001, one-way ANOVA. **D** qRT-PCR was used to quantify IL-1β, TNF-α, and IL-6 mRNA in lung tissue from each treatment group, with values normalized to β-actin mRNA (*n* = 5). Columns show the mean ± SD of multiple independent experiments; **P < 0.01, one-way ANOVA.

Immediately upon establishment of the BPD model, a robust inflammatory response was observed, manifesting as a progressive increase in total inflammatory cell counts over time (Fig. 3A). This response was markedly attenuated in GSDMD^−/−^ mice, with statistically significant differences versus WT littermates. Quantification of alveolar macrophages (AMs) recovered from lung tissue revealed a significant reduction in GSDMD^−/−^ animals (Fig. 3B). BALF cytokine profiling demonstrated pronounced elevations of pro-inflammatory mediators (IL-1β, IL-6, and TNF-α) in BPD-affected mice, whereas levels of these cytokines were significantly lower in GSDMD^−/−^ mice than in WT controls (Fig. 3C). Concurrently, anti-inflammatory cytokines (IL-4 and IL-10) were up-regulated in BPD lungs as a compensatory mechanism; GSDMD deletion further amplified this anti-inflammatory arm, thereby mitigating overall inflammation (Supplementary Fig. S1A). qRT-PCR of lung tissue corroborated these findings: BPD mice exhibited elevated mRNA expression of IL-1β, TNF-α, IL-6, IL-4, and IL-10, whereas GSDMD^−/−^ mice displayed a reciprocal shift—down-regulation of pro-inflammatory transcripts and sustained up-regulation of anti-inflammatory IL-4 and IL-10 (Fig. 3D and Supplementary Fig. S1B). Collectively, the data demonstrate that GSDMD deletion re-calibrates macrophage activation toward an anti-inflammatory phenotype and thereby mitigates BPD-associated pulmonary inflammation.

### GSDMD knockout promotes macrophage polarization from M1 to M2

**Fig. 4:**
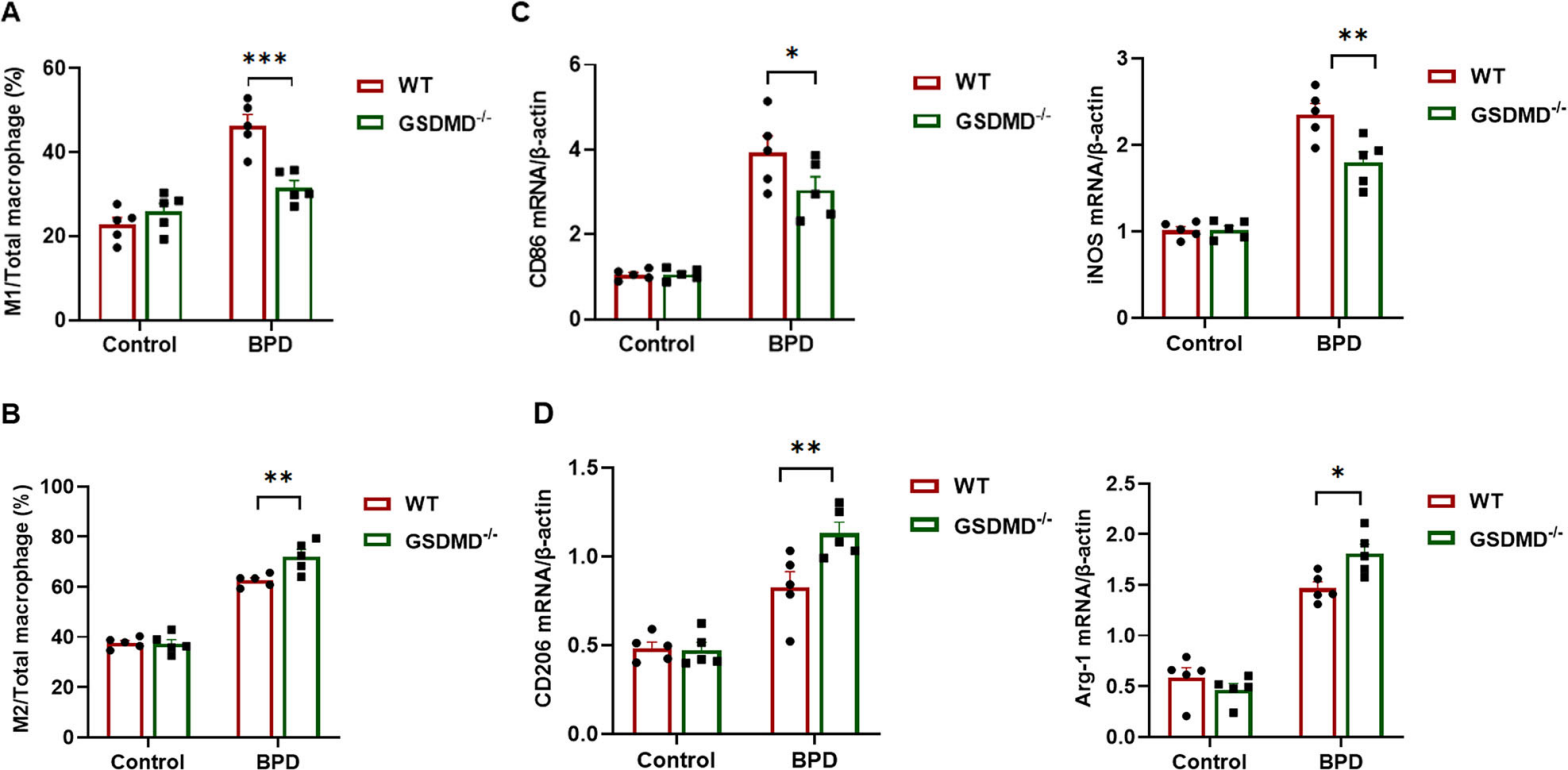
GSDMD knockout promotes macrophage polarization from M1 to M2. **A** AMs were identified using FITC-conjugated anti-mouse CD86 and APC-conjugated anti-mouse F4/80 antibodies. The proportion of M1 macrophages relative to total macrophages was quantified by flow cytometry. Columns represent the mean ± SD of multiple independent experiments (*n* = 5 per group); ***P < 0.001, one-way ANOVA. **B** M2 macrophages were identified with PE-Cy5-conjugated anti-mouse CD206 and APC-conjugated anti-mouse F4/80 antibodies, and their percentage relative to total macrophages was analyzed analogously. Columns depict the mean ± SD of multiple independent experiments (*n* = 5 per group); **P < 0.01, one-way ANOVA. **C** qRT-PCR was employed to measure the mRNA levels of M1 macrophage markers (CD86, iNOS) in lung tissue, normalized to β-actin as the internal control (*n* = 5 per group). Columns show the mean ± SD of multiple independent experiments; *P < 0.05, **P < 0.01, one-way ANOVA. **D** qRT-PCR was used to determine the mRNA levels of M2 macrophage markers (CD206, Arg-1) in lung tissue, normalized to β-actin as the internal control (*n* = 5 per group). Columns display the mean ± SD of multiple independent experiments; *P < 0.05, **P < 0.01, one-way ANOVA.

The impact of GSDMD knockout on AMs polarization in experimental BPD mice was investigated using flow cytometry. Flow-cytometric analysis revealed an elevated total macrophage infiltration in the BALF of BPD-affected pups, primarily characterized by M1 macrophage markers (CD11b^+^F4/80^+^CD86^+^). Nevertheless, the rise in the M1-to-total macrophage ratio observed during BPD was notably mitigated by GSDMD knockout, as depicted in Fig. 4A. This alteration was further distinguished by an escalation in the M2-to-total macrophage ratio in the context of GSDMD knockout, as illustrated in Fig. 4B.

To explore the impact of GSDMD knockout on macrophage polarization, we conducted in vitro experiments utilizing BMDMs. Untreated BMDMs were exposed to LPS or IL-4 to prompt differentiation into M1-like macrophages (M1, F4/80^+^CD86^+^) or M2-like macrophages (M2, F4/80^+^CD206^+^). The transition from M1 to M2 polarization in the knockout of GSDMD was apparent, as indicated by distinct macrophage phenotype markers (Fig. 4C). This observation suggests that GSDMD plays a role in regulating macrophage polarization from the pro-inflammatory M1 phenotype to the anti-inflammatory M2 phenotype during inflammatory responses.

In line with the observed alterations in macrophage phenotype, we detected notable decreases in the synthesis of pro-inflammatory cytokines in macrophages upon GSDMD knockout. Conversely, there was a marked increase in the release of anti-inflammatory cytokines (Fig. 4D). Taken together, our results indicate that the knockout of GSDMD could mitigate pulmonary damage associated with BPD through the modulation of macrophage polarization and cytokine equilibrium, presenting a promising therapeutic avenue for BPD treatment.

### GSDMD knockout inhibits pyroptotic stimuli-induced IL-1β release in macrophages

**Fig. 5:**
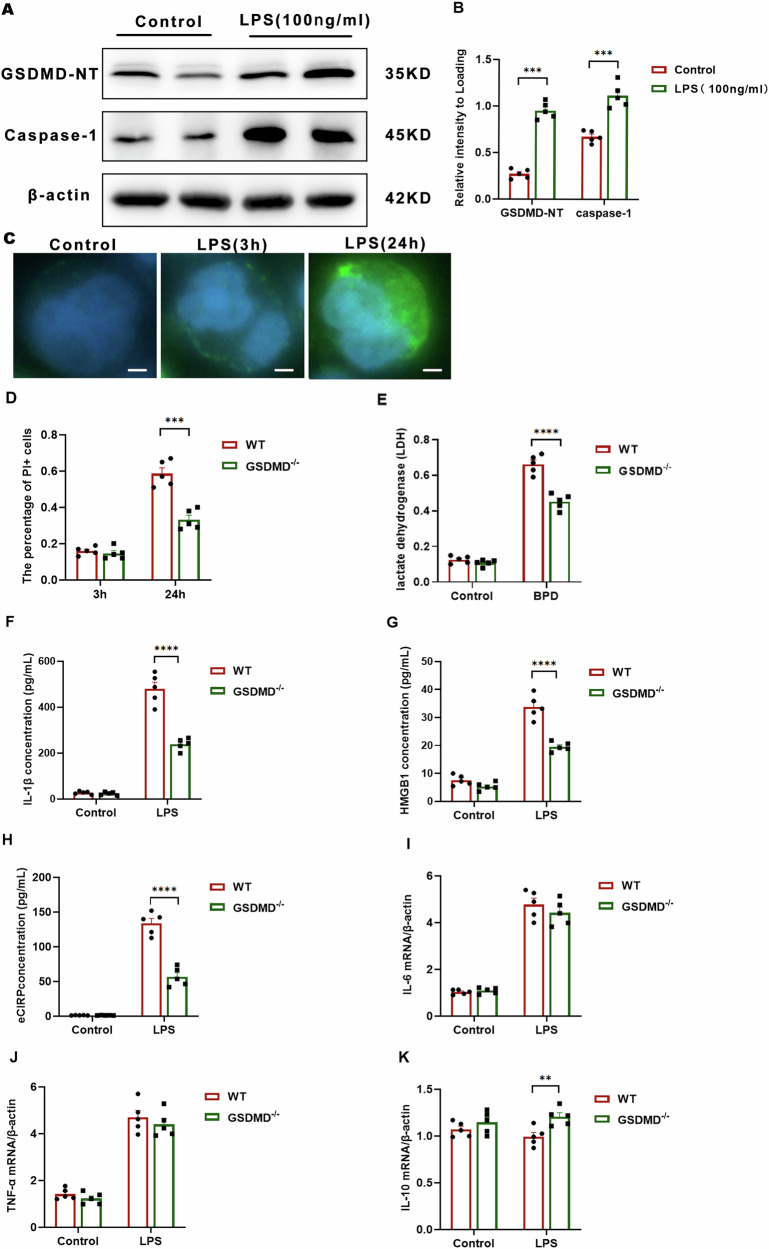
GSDMD knockout inhibits pyroptotic stimuli-induced IL-1β release in macrophages. **A** Immunoblotting was performed to assess GSDMD-NT and full-length Caspase-1 in BMDMs across treatment groups. Representative blots from three independent experiments with consistent results are shown. **B** Bands were quantified using ImageJ. Data are presented as mean ± SD from three biologically independent samples; ***P < 0.001, two-way ANOVA. **C** BMDMs were stimulated with LPS (100 ng mL⁻¹) and, at indicated time points, immunostained with an anti-GSDMD monoclonal antibody and counterstained with DAPI (blue). Pore formation was defined as the percentage of cells displaying ≥ 3 membrane blebs/bubbles within 30 min of LPS + ATP treatment. Images are representative of at least three independent experiments with random sampling; scale bar = 10 μm. **D** The proportion of PI-positive BMDMs was determined at each time point, with ≥ 200 cells evaluated per sample. Data are mean ± SD from multiple biologically independent experiments; ***P < 0.001, one-way ANOVA. **E** Macrophage death was evaluated by LDH release, expressed as the percentage of LDH release in the supernatant relative to total cellular LDH. Bars represent mean ± SD from multiple independent experiments; ****P < 0.0001, one-way ANOVA. **F**–**H** Secretion of IL-1β, HMGB1 and eCIRP by BMDMs was quantified by ELISA. Each group comprised five replicates from three biologically independent samples. Data are mean ± SD; ****p < 0.0001, one-way ANOVA. **I**–**K** Pulmonary mRNA levels of IL-6, TNF-α and IL-10 were normalized to β-actin and measured by qRT-PCR in the BPD group (*n* = 5) and control group (*n* = 5). Data are mean ± SD; **P < 0.01, one-way ANOVA.

We investigated the ability of LPS to trigger pyroptotic activation in macrophages and the subsequent GSDMD-dependent release of pro-inflammatory cytokines. Upon LPS stimulation, BMDMs exhibited markedly elevated Caspase-1 expression, generation of the GSDMD-NT, and appearance of cleaved Caspase-1, indicating robust GSDMD activation (Fig. 5A, B and Supplementary Fig. S2A, S2B). Consistent with GSDMD cleavage, we observed reduced Caspase-1 activation (P20) in GSDMD^−/−^ macrophages following LPS/nigericin treatment. To define the subcellular redistribution of GSDMD after LPS challenge, we performed immunofluorescence staining of BMDMs (Fig. 5C). Under resting conditions, GSDMD was predominantly localized in the cytoplasm and nucleus. Following LPS exposure, GSDMD translocated to the plasma membrane, consistent with N-terminal cleavage, oligomerization, and pore formation (Fig. 5C). We evaluated plasma membrane integrity using the membrane-impermeable dye PI. After exposure to LPS, a gradual increase in PI-positive cells was observed. Importantly, the percentage of PI-positive cells in GSDMD^−/−^ BMDMs was significantly lower than that in WT BMDMs at all time points beyond 3 h (Fig. 5D). Macrophage lysis was assessed through the quantification of LDH release. A significant elevation in LDH release from WT BMDMs was observed following LPS stimulation over time. Notably, this release was decreased by 30% at the 12-h time point after LPS treatment in GSDMD^−/−^ BMDMs, indicating that the knockout of GSDMD alleviates macrophage lytic cell death (Fig. 5E).

To investigate the role of GSDMD pores in mediating the release of non-lytic inflammatory mediators from macrophages upon exposure to LPS, we measured IL-1β, high mobility group box 1 (HMGB1) and the extracellular levels of cold-inducible RNA-binding protein (eCIRP). All of which are known to be discharged through GSDMD pores (Fig. 5F–H). Our findings revealed that the treatment of WT BMDMs with LPS ex vivo significantly increased the secretion of IL-1β, HMGB1, and eCIRP. In contrast, GSDMD^−/−^ BMDMs showed significant reductions in the release of inflammatory factors upon LPS stimulation (Fig. 5F–H). Notably, GSDMD knockout did not completely inhibit the production of IL-6, tumor TNF-α, and IL-10 (Fig. 5I–K), suggesting that GSDMD-independent pathways may contribute to inflammation.

### GSDMD knockout boosts macrophage pathogen-killing capacity

**Fig. 6:**
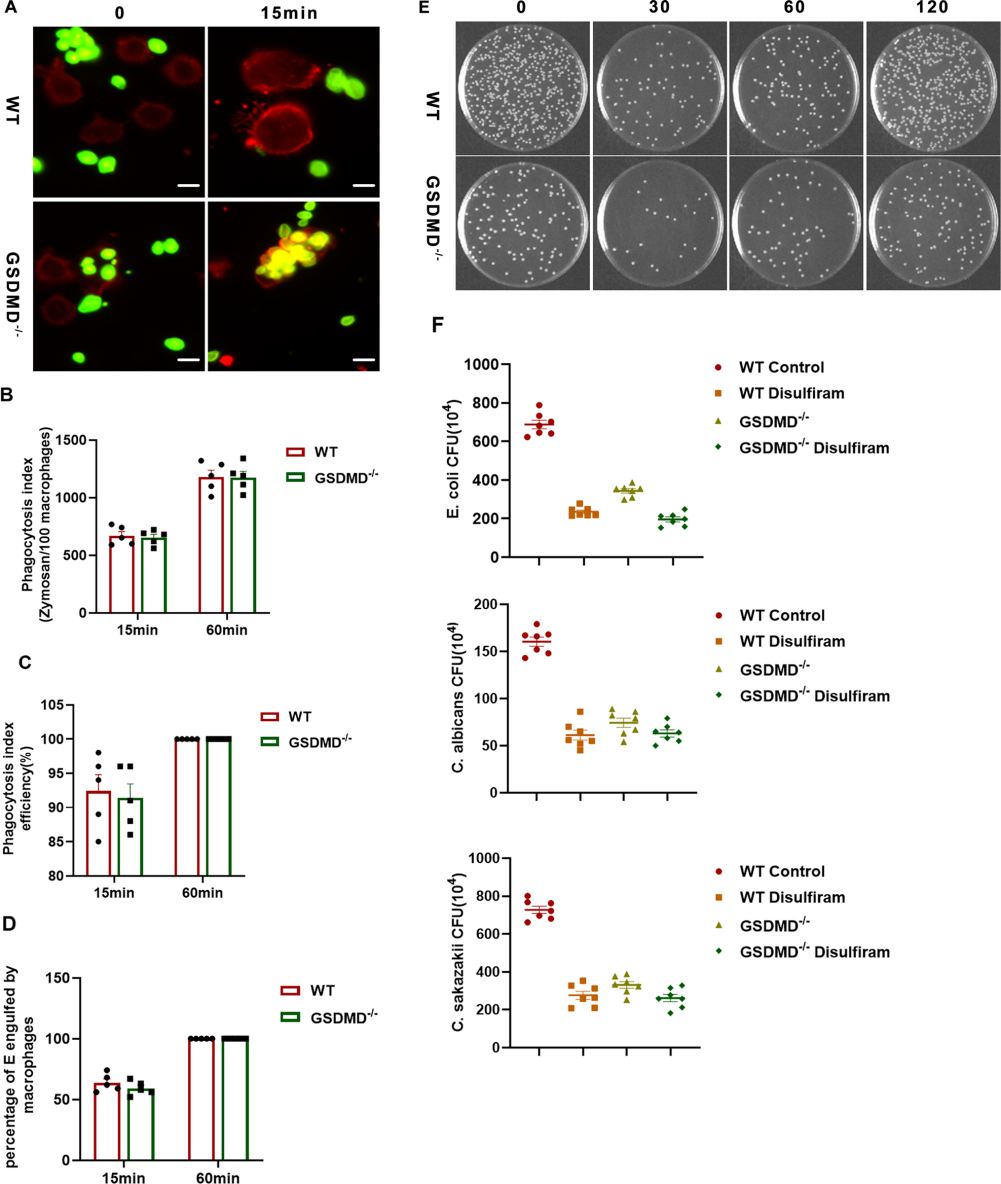
GSDMD knockout boosts macrophage pathogen-killing capacity. **A** BMDMs from WT and GSDMD⁻^/^⁻ mice were subjected to in vitro phagocytosis assays using mouse-serum-opsonized, FITC-labeled zymosan particles. Extracellular fluorescence was quenched with trypan blue; representative images are shown (scale bar, 10 µm). **B** The phagocytic index is defined as the number of bioparticles engulfed per 100 macrophages and serves as a quantitative measure of phagocytic capacity. **C** Phagocytic efficiency is expressed as the percentage of BMDMs that internalized at least one bioparticle; a minimum of 200 cells was analyzed per sample. Data are presented as mean ± SD from five biologically independent samples per group. **D** The percentage of bioparticles ingested by macrophages was likewise quantified, with data derived from five biologically independent samples per group (mean ± SD). **E** WT and GSDMD⁻^/^⁻ BMDMs were co-cultured with opsonized, live Escherichia coli at 37 °C in an in vitro bactericidal assay. Serial dilutions were plated onto Luria–Bertani agar, and colony counts were enumerated at indicated time points. **F** Antimicrobial activity of WT versus GSDMD⁻^/^⁻ BMDMs under the indicated treatments is depicted (*n* = 7). Medium alone served as the control. Results against **E** coli, Candida albicans, and Cronobacter sakazakii are shown, with data expressed as mean ± SD.

Macrophages defend immunity via phagocytosis and antibacterial activity; we examined how GSDMD knockout affects their pathogen clearance. In a comprehensive phagocytosis assessment, both WT and GSDMD^−/−^ BMDMs efficiently internalized FITC-labeled COZ particles, exhibiting comparable phagocytic indices, efficiency, and percentage of particle uptake (Fig. 6A–D). To further elucidate the involvement of macrophage pyroptosis in bacterial clearance, a bacterial killing assay was performed. WT macrophages demonstrated a 25% reduction in colony-forming units (CFUs), indicative of effective suppression of E. coli growth and survival. Macrophages from GSDMD^−/−^ exhibited a notable increase in their capacity to inhibit bacterial proliferation, leading to a reduction exceeding 50% (Fig. 6E). This heightened efficacy was also evident in the case of C. albicans and C. sakazakii, pathogens associated with severe infant infections (Fig. 6F). In essence, GSDMD knockout boosts macrophage resilience, hinders pathogen evasion, particularly by C. albicans, and thereby reinforces their ability to eliminate these microorganisms.

### ROS drive macrophage pyroptosis via GSDMD N-terminal cleavage and pore formation

**Fig. 7:**
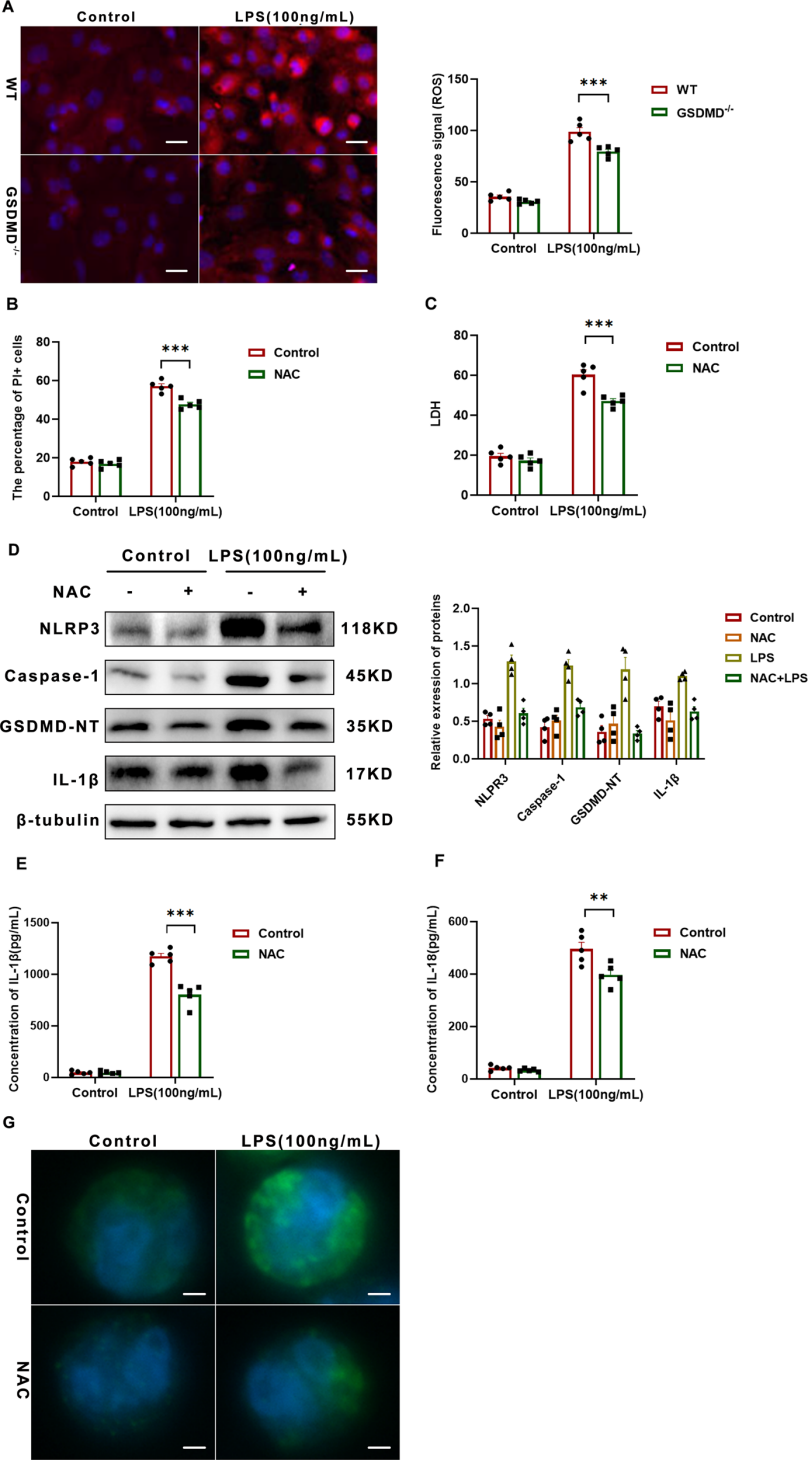
ROS drive macrophage pyroptosis via GSDMD N-terminal cleavage and pore formation. **A** Fluorescence micrographs depict superoxide anion (O₂•⁻) detection in formalin-fixed lung sections using DHE, which is oxidized by O₂•⁻ to yield a red fluorescent product; scale bar = 50 μm. Images are representative of at least five mice per treatment group. The right panel shows quantitative analysis of DHE-derived fluorescence intensity using ImageJ-1.46r, performed on a minimum of three independent coverslips per condition; data are presented as mean ± SD; ***P < 0.001 (one-way ANOVA). **B** The percentage of PI-positive BMDMs in the indicated treatment groups was determined; at least 200 cells were counted per sample. Data are presented as mean ± SD; ***P < 0.001 (one-way ANOVA). **C** Macrophage death was assessed by LDH release assay. Relative LDH release is expressed as the percentage of LDH release in the culture supernatant relative to total cellular LDH release, serving as an index of cytotoxicity. Bars represent mean ± SD from multiple independent experiments; ***P < 0.001 (one-way ANOVA). **D** Lung tissues from neonatal pups in the indicated treatment groups were analyzed by Western blot for NLRP3, Caspase-1, GSDMD-NT, and IL-1β expression. Representative blots from three independent experiments are shown. The right panel presents quantification of NLRP3, Caspase-1, GSDMD-NT, and IL-1β levels normalized to the loading control (*n* = 4) and expressed as mean ± SD; *P < 0.05, **P < 0.01, ***P < 0.001, ****P < 0.0001 (one-way ANOVA). **E** IL-1β secretion by BMDMs in the indicated treatment groups was measured by enzyme-linked immunosorbent assay (ELISA; n = 5). Data are presented as mean ± SD; ***P < 0.001 (one-way ANOVA). **F** IL-18 secretion by BMDMs in the indicated treatment groups was similarly quantified by ELISA (*n* = 5); data are presented as mean ± SD; **P < 0.01 (one-way ANOVA). **G** BMDMs from the indicated treatment groups were immunostained with an anti-GSDMD monoclonal antibody and counterstained with DAPI (blue); scale bar = 10 μm. Images are representative of at least three independent experiments.

It is well established that macrophages typically eliminate internalized bacteria by utilizing ROS. Additionally, ROS plays a crucial role in modulating inflammatory pathways, which are tightly controlled by the NLRP3 inflammasome. In our study, we evaluated ROS levels in BMDMs under LPS-induced stress by quantifying the fluorescence intensity of 2′,7′-dichlorofluorescin (DCF). As illustrated in Fig. 7A, LPS significantly increased endogenous ROS generation compared with the negative-control group. Conversely, GSDMD^−/−^ BMDMs displayed reduced ROS levels (Fig. 7A).

Pre-treatment with the ROS scavenger N-acetyl-L-cysteine (NAC) significantly attenuated cell death, as evidenced by reduced PI staining and diminished LDH release compared with vehicle-treated controls (Fig. 7B, C). Subsequent immunoblot analyses revealed that NAC consistently down-regulated the protein levels of NLRP3, full-length GSDMD, pro-caspase-1, pro-IL-1β, and the cleaved forms of GSDMD-NT and Caspase-1 P20 (Fig. 7D and Supplementary Fig. S3A and S3B). Moreover, NAC markedly suppressed the secretion of mature IL-1β and IL-18 (Fig. 7E, F). Collectively, these findings demonstrate that ROS inhibition partially abrogates NLRP3 inflammasome activation, cytokine release, and pyroptotic cell death, underscoring an essential role for ROS in macrophage pyroptosis.

To investigate the influence of ROS on GSDMD activity, we utilized immunofluorescence to analyze GSDMD localization for visualizing GSDMD pore formation (Fig. 7G). Upon LPS treatment without any inhibitors, GSDMD staining was notably present in both the cytosol and plasma membrane, resulting in the formation of distinct pyroptotic bubbles. Conversely, treatment with the ROS inhibitor NAC markedly decreased GSDMD staining on the cell membrane and the generation of pyroptotic bubbles, thereby affirming the crucial involvement of ROS in GSDMD activation.

### Disulfiram mitigates the severity of experimental BPD

**Fig. 8:**
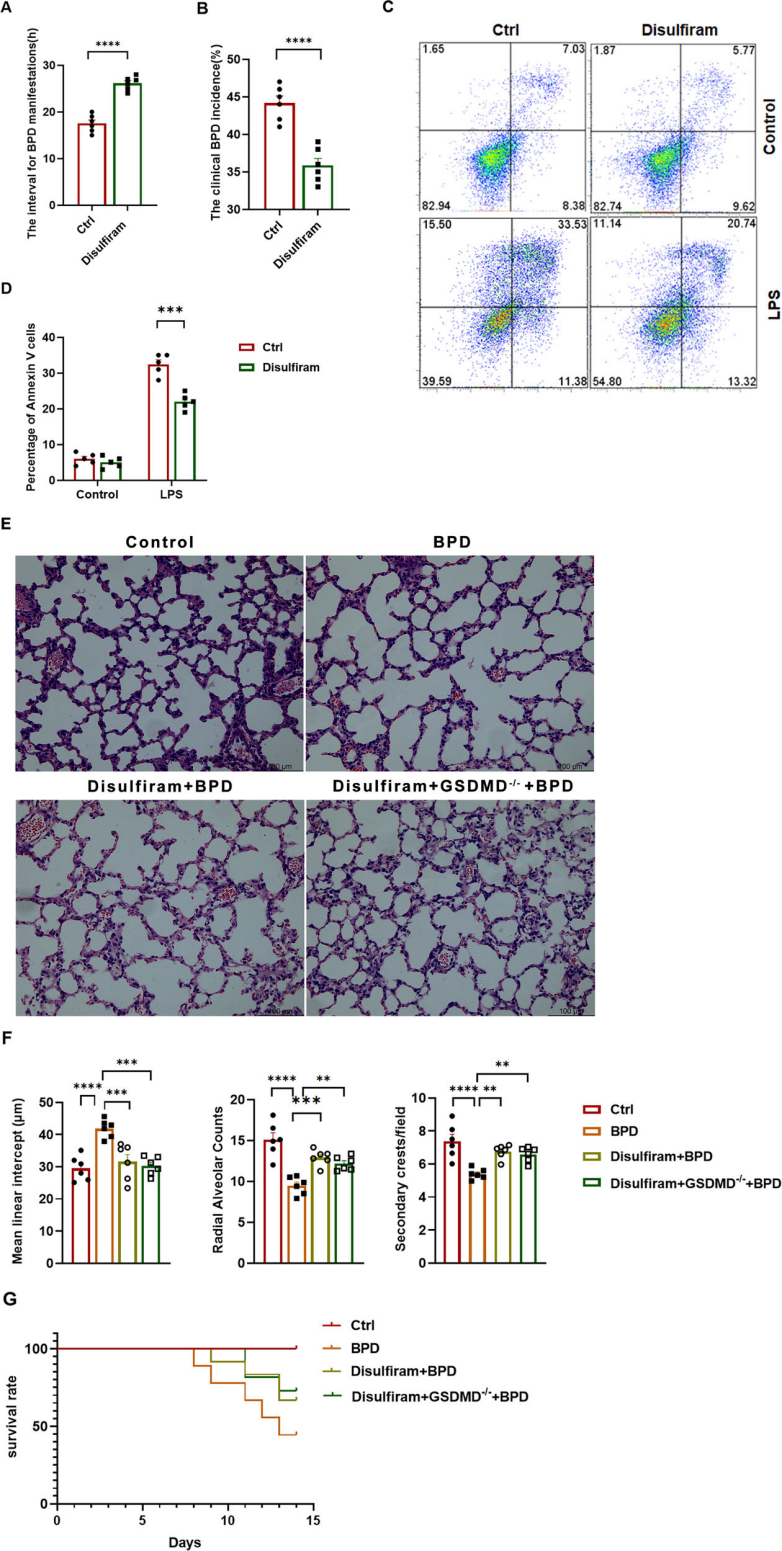
Disulfiram mitigates the severity of experimental BPD. **A** The time interval from the initiation of the BPD regimen to the onset of symptoms is depicted in days. Bars represent mean values for each group (*n* = 6 mice); data are expressed as mean ± SD; ****P < 0.0001 (one-way ANOVA). **B** The incidence of BPD in neonatal pups across the indicated treatment groups is presented. Bars denote group means (*n* = 6 mice); data are shown as mean ± SD; ****P < 0.0001 (one-way ANOVA). **C** Flow cytometry was employed to assess C D11c and F4/80 expression in BMDMs from the indicated treatment groups. The percentages of viable cells (region R1), early apoptotic cells (region R2), and late apoptotic/necrotic cells (regions R3 and R4) are indicated. **D** A schematic summary is provided on the right. **E** Representative H&E staining of the left lungs from mice with experimental BPD is displayed. Images are representative of at least three independent replicate coverslips per condition; scale bar = 100 µm. **F** Representative H&E-stained lung sections from each treatment group (*n* = 6 per group, 12 fields per animal) were subjected to morphometric analysis to evaluate alveolar development, including MLI, RAC and the number of secondary crests per field. Scale bar = 100 µm. Bars represent the averages of three independent experiments; values are presented as mean ± SD; **P < 0.01, ***P < 0.001, ****P < 0.0001 (one-way ANOVA). **G** Kaplan–Meier survival analysis comparing the survival of experimental BPD mice among different treatment groups (*n* = 9 mice per group).

Disulfiram is a recently identified covalent inhibitor of GSDMD pore formation [17, 23], and it exhibits therapeutic promise in experimental BPD by blocking GSDMD-mediated pore formation and attenuating inflammatory responses. In this preclinical BPD model, intraperitoneal administration of 50 mg/kg disulfiram delayed symptom onset, reduced disease incidence (Fig. 8A, B), and prolonged survival of BMDMs (Fig. 8C, D). Histopathological examination revealed marked improvement in pulmonary architecture following disulfiram treatment (Fig. 8E, F), with significant mitigation of BPD-associated morphological alterations. Survival analyses further demonstrated that 50 mg/kg disulfiram increased overall survival in BPD mice, whereas this protective effect was absent in GSDMD-knockout animals (Fig. 8G). The divergent outcomes between WT and GSDMD^−/−^ cohorts underscore the mechanism-specific therapeutic action of disulfiram in this context.

## Discussion

Our study shows that AMs undergo pyroptosis via the GSDMD pathway, releasing pro-inflammatory mediators linked to BPD pulmonary damage. Blocking GSDMD signaling shifts macrophages to the anti-inflammatory M2 phenotype, protecting against lung injury and suggesting a new BPD treatment strategy [24]. This is the first study to clarify GSDMD’s role in macrophage function and BPD pathogenesis [25]. In hyperoxia-exposed BPD mice, activated macrophages trigger alveolar epithelial apoptosis, disrupt lung architecture, and impair fibroblasts and endothelial cells, leading to matrix remodeling and compromised vascular development. Macrophage pyroptosis, a lytic form of cell death and a key innate immune mechanism [26, 27], has an unclear role in BPD pathogenesis.

GSDMD is recognized as a crucial mediator of pyroptosis, a form of programmed cell death triggered by danger signals from pathogens [16, 21]. Activation of GSDMD via cleavage by inflammatory C aspases results in the creation of pores that compromise cell membrane integrity, ultimately inducing pyroptosis [23]. This inflammatory process has been linked to the initial shock stages observed in systemic hyperinflammatory reactions [28, 29]. Prior studies have shown that GSDMD knockout leads to enhanced lung alveolarization and vascularization, decreased lung inflammation, and diminished cell death when exposed to hyperoxia [30]. Surprisingly, GSDMD⁻/⁻ lungs exhibited an even lower GSH/GSSG ratio than WT-BPD lungs. This may reflect compensatory up-regulation of alternative oxidative pathways (e.g., Nrf2-independent glutathione consumption) or heightened recycling of GSSG; further studies are warranted to dissect this redox reprogramming. In this investigation, we observed the activation of the NLRP3 inflammasome, Caspase-1, and GSDMD, alongside a notable increase in the pro-inflammatory cytokine IL-1β, in the context of lung injury associated with BPD. A marked elevation in GSDMD expression in lung tissue was detected post-BPD onset, concomitant with pulmonary injury in BPD pathogenesis. Correspondingly, GSDMD^−/−^ mice exhibited significantly enhanced survival rates and diminished myocardial injury in the setting of lethal bacterial sepsis. GSDMD deletion directly abrogates pore formation, thereby curtailing ROS amplification, lowering the ROS-mediated priming threshold for NLRP3 inflammasome activation, and diminishing NLRP3-ASC oligomerization. Consequently, Caspase-1 activation and IL-1β maturation/secretion are markedly suppressed. Whereas IL-1β release is sharply attenuated, IL-10 and TNF-α levels remain unaltered. By contrast, TNF-α and IL-6 are chiefly transcribed via the NF-κB pathway, which remains intact in GSDMD⁻/⁻ macrophages. Thus, GSDMD knockout functionally uncouples inflammasome-dependent IL-1β processing from broader TLR–NF-κB signaling, accounting for the sustained TNF-α/IL-6 output.

This study demonstrates that GSDMD is up-regulated and activated in a murine model of BPD. Genetic ablation of GSDMD markedly attenuates excessive inflammatory responses and pulmonary dysfunction, underscoring the pivotal role of pyroptosis and inflammatory activation in BPD progression and establishing a foundation for future targeted therapeutic investigations. This observation aligns with findings from studies on mice lacking other inflammasome constituents, such as NLRP3, Caspase-1/11, and ASC, which are integral to the upstream signaling cascades of GSDMD. These investigations similarly reported suppression of IL-1β production and bacterial sepsis [31–35].

In BPD neonatal mice, circulating monocytes infiltrate the pulmonary lamina propria and differentiate into lung macrophages [36]. We provide the first evidence that GSDMD governs stress-induced macrophage pyroptosis and drives BPD pathogenesis; its deletion reprograms macrophage trafficking and cytokine signaling, unveiling a complex GSDMD–macrophage–innate immunity axis. In vivo, GSDMD knockout markedly curbs hyperoxia-triggered macrophage infiltration and lung inflammation, offering a novel therapeutic target against hyperoxic lung injury. Our findings align with emerging evidence that persistent neutrophil activation contributes to BPD progression through NETosis and IL-17-mediated inflammation [37]. Beyond BPD, GSDMD-driven pyroptosis has been implicated in neonatal necrotising enterocolitis (NEC), where intestinal epithelial GSDMD activation exacerbates mucosal injury [38]. In contrast to the lung, NEC lesions show persistent TNF-α elevation despite GSDMD inhibition, highlighting tissue-specific inflammatory circuits. These parallels underscore the therapeutic potential and the context-dependency, of targeting GSDMD in neonatal inflammatory disorders. Notably, while phagocytosis of zymosan particles (a mannose receptor-mediated process) remained unchanged, GSDMD^−/−^macrophages exhibited significantly enhanced killing of internalized E. coli. This bactericidal advantage is independent of phagocytic capacity but attributable to:

(i) Enhanced ROS-dependent mechanisms: Elevated mitochondrial ROS production synergizes with NADPH oxidase-derived ROS to amplify pathogen killing downstream of bacterial internalization [39];
(ii) Preserved lysosomal function: Unruptured lysosomes in GSDMD^−/−^ cells promote efficient delivery of antimicrobial enzymes to phagosomes [40];
(iii) Prolonged macrophage viability: Inhibited pyroptosis extends macrophage lifespan, enabling sustained antimicrobial activity.

The reduced macrophage population in GSDMD^−/−^ mice correlates with diminished inflammatory reactions. Macrophages display diverse phenotypes influenced by complex networks affecting their maturation, stimulation, and function [41]. The traditional M1/M2 dichotomy is now considered outdated [42]. Pro-inflammatory M1 macrophages drive pathological inflammation in BPD by secreting cytokines [42], and strategies to reduce their infiltration have shown promise in mitigating inflammatory disorders like sepsis [43]. Ams from GSDMD^−/−^ mice are smaller and produce fewer inflammatory cytokines, indicating immaturity. These macrophages also show stronger anti-inflammatory properties compared to WT mice, likely due to maintained anti-inflammatory traits and enhanced M2 polarization [15]. This suggests that GSDMD^−/−^ macrophages could be therapeutically valuable in managing inflammatory diseases like BPD. Additionally, GSDMD is involved in monocyte-to-macrophage differentiation [44].

Previous studies have shown that BPD-induced damage enhances the function of chemokines and their receptors (e.g., CXCL3, CCR1, CCR2, CCR5), which direct immune cells to sites of infection or injury [45, 46]. Our study found reduced infiltration of monocytes and macrophages in the lungs of GSDMD^−/−^ mice, along with lower expression of surface markers, highlighting GSDMD’s key role in immune cell recruitment and inflammation [47]. The observed reduction in neutrophil infiltration in GSDMD⁻^/^⁻ mice may reflect disrupted inflammasome-dependent neutrophil recruitment, particularly through impaired IL-1β-mediated CXCL1/2 production.

Notably, GSDMD activity is further modulated by post-translational modifications such as palmitoylation, which promotes its membrane trafficking and pore formation [16]. Given emerging evidence for palmitoylation in regulating GSDMD, future studies should investigate whether hyperoxia alters this modification in macrophages and whether palmitoylation inhibitors synergize with disulfiram in BPD treatment. While our study focuses on genetic and pharmacological inhibition of GSDMD, future work should explore whether palmitoylation contributes to its pathogenic role in BPD. While macrophages are the primary focus of this study, we observed that GSDMD deficiency also reduced neutrophil infiltration (Fig. 4E) [37]. This may have particular clinical relevance, as recent single-cell studies identify pathogenic IL-17A+ neutrophil subsets in BPD patients [37, 48], and demonstrate that neutrophil-derived proteases promote alveolar simplification [49]. Our findings suggest GSDMD inhibition could simultaneously target both the macrophage and neutrophil arms of inflammatory lung injury [37].

## Conclusion

In summary, our study demonstrates that GSDMD knockout attenuates the inflammatory response in BPD by reducing inflammatory mediator release and macrophage-driven lung injury. It simultaneously mitigates M1 macrophage-associated inflammation, thereby providing significant protection against BPD. These findings position GSDMD inhibition as a promising strategy to break the vicious cycle of inflammation and impaired lung development in prematurity.

## Materials and methods

### Experimental BPD model

All experiments were approved by the animal care and use committee of Chongqing Medical University. C57BL/6 mice were provided by Dr Wenli Han (Experimental Animal Center, Chongqing Medical University), and GSDMD knockout (GSDMD⁻/⁻) mice were kindly donated by Dr Hongbo Luo (Boston Children’s Hospital).

Post-natal day 1 C57BL/6 pups (mean weight 0.96 ± 0.10 g) were subjected to a well-validated protocol to induce BPD-like pathology. Within 24 h of birth, pups derived from at least three litters were randomized by sealed-envelope assignment into normoxia or hyperoxia chambers, ensuring equal sex distribution (four males and four females per group). Sample size was pre-calculated using G*Power 3.1 (α = 0.05, power = 0.80, effect size = 1.2 based on pilot MLI data), indicating a minimum of six pups per group; to account for ~10% hyperoxia-related mortality, eight pups were enrolled per group. The 14-day exposure window was selected to model the murine saccular-to-alveolar transition, encompassing the entire secondary septation period (post-natal days 14–21) and reliably reproducing the cardinal human BPD hallmarks of chronic alveolar simplification and impaired pulmonary vascular development; exposures ≤7 days elicit only acute inflammation. Oxygen concentration was continuously monitored; male and female pups were evenly distributed, and nursing dams were rotated between hyperoxia and normoxia litters every 24 h. All experimental procedures and outcome assessments were performed by investigators blinded to group allocation to minimize bias in data collection and analysis.

Throughout the study, clinical incidence of BPD, body-weight gain, disease onset, and survival were systematically recorded. To evaluate disulfiram efficacy, pups received daily intraperitoneal injections of disulfiram (Biochempartner, Shanghai; 50 mg kg⁻¹) for 7 consecutive days beginning on day 14 of hyperoxia exposure. Disulfiram was prepared fresh daily in 5% DMSO/corn oil based on established protocols for GSDMD inhibition [5]. This dose translates to a human equivalent daily dose of 284 mg and falls within the clinically approved range of 125–500 mg day⁻¹ for alcohol-dependence therapy.

At predetermined time points, pups were euthanized with sodium pentobarbital. Lungs were harvested for subsequent analyses. A blinded pathologist performed morphometric analyses, including mean linear intercept (MLI), secondary crest counts, and radial alveolar counts (RAC), as previously described [3, 4]. The experimental timeline is illustrated in Fig. 1.

### Glutathione (GSH) and glutathione disulfide (GSSG) assessment

Snap-frozen lung tissues were homogenized in sterile saline and centrifuged. The resulting supernatant was then subjected to analysis to quantify levels of reduced GSH and GSSG, following established protocols [5, 11]. Twenty microliters of the supernatant was analyzed using a GSH and GSSG assay kit (S0053, Beyotime). The GSSG/GSH ratio served as a marker of oxidative stress. Data were derived from three replicates and are presented as nanomoles per milligram of protein (nmol/mg of protein).

### Histological analysis

Lung tissues from mice were fixed in 4% paraformaldehyde overnight, then embedded in paraffin, and sectioned at 5 μm thickness for subsequent staining with hematoxylin and eosin (H&E). Histological images were captured using a Nikon Eclipse E100 microscope. Five samples per group were randomly chosen, and three different sections were obtained from the maximum lung cross-sectional area of each sample. Three non-overlapping fields of view were randomly selected from each section for imaging and analysis. Image processing and morphological evaluation of lung tissues were conducted using ImageJ software. Statistical parameters assessed included MLI, RAC, and secondary crests per field.

### Assessments of superoxide dismutase (SOD) and malondialdehyde (MDA) in lung tissues

Lung tissues, freshly dissected, were processed following established protocols [17]. The samples were homogenized, and the resulting supernatants were used to assess SOD and MDA levels using commercially available kits according to the manufacturer’s guidelines. Carbonyl protein levels were measured using a Protein Carbonyl Colorimetric Assay Kit (Cayman Chemical Company, USA). All results were normalized to protein concentration.

### Immunohistochemical analysis

Immunohistochemical analysis was conducted on paraffin-embedded lung tissue samples from mice. The 5-μm-thick sections underwent deparaffinization, rehydration, and washing. A rabbit polyclonal primary antibody against mouse GSDMD was applied and incubated overnight at the appropriate dilution in blocking buffer. Subsequently, the sections were treated with secondary antibodies, and the immunoreactivity was visualized using a DAB Substrate Kit (Thermo Fisher Scientific, Waltham, MA). The slides were counterstained with hematoxylin and examined under a Nikon 55I microscope (Nikon, Tokyo, Japan).

### BMDMs isolation and stimulation

BMDMs were isolated from 8-week-old adult mice by flushing femurs and tibias, followed by 6-day culture in medium containing 20 ng/mL M-CSF to yield mature macrophages [5]. Flow cytometry confirmed >95% F4/80⁺ cells. The complete gating scheme: singlets → viable CD45⁺ cells → F4/80⁺CD11b⁺ macrophages → M1 (CD86⁺CD206⁻) and M2 (CD206⁺CD86⁻) subsets. The BMDMs were seeded onto poly-L-lysine-coated 12-well plates. For pyroptosis induction, LPS (100 ng/mL) was transfected into macrophages using Lipofectamine 2000 for 6 h, followed by nigericin (5 μM, 1 h) treatment. LPS concentrations were context-optimized: 100 ng/mL for in vitro transfection (to assess pyroptosis) and 10 μg/g for in vivo challenge (to ensure consistent lung injury). For pharmacological treatments, disulfiram (0–1 μM) was added to the culture medium 12 h before C. albicans infection or LPS priming.

### Quantitative real-time PCR (qRT-PCR)

Total RNA was extracted from fresh lung tissues using TRIzol (Invitrogen). One microgram of RNA was reverse-transcribed with oligo-dT(18) and SuperScript II reverse transcriptase (Invitrogen) and amplified on an ABI 7300 system (Applied Biosystems). Gene-specific primers were synthesized by Thermo Fisher Scientific. Relative gene expression was calculated by the 2^−ΔΔCt^ method and normalized to β-actin. The primer sequences are listed in Supplementary Material 1.

### Dihydroethidium staining for superoxide analysis

Intracellular superoxide levels were quantified following a previously established protocol [3, 4]. BMDMs were cultured in 12-well plates and treated with dihydroethidium (DHE, 5 μM; Thermo Fisher Scientific) for 30 minutes at 37 °C. Subsequently, the cells were counterstained with 4′,6-diamidino-2-phenylindole (DAPI; Sigma-Aldrich) for nuclear staining. Reactive oxygen species (ROS), specifically superoxide anions (O_2_•^–^), were visualized at an excitation wavelength of 535 nm using a Leica TCS SP5 confocal microscope (Leica Microsystems). Quantitative analysis of confocal images was performed using ImageJ software (National Institutes of Health), with twenty high-power fields evaluated per tissue slide.

### Endotoxin assay

LPS levels were quantified using the Mouse Lipopolysaccharide(LPS) ELISA Kit from Jianglai Biotechnology, performed strictly according to the manufacturer’s instructions. The assay’s analytical range is 0.156–10 ng mL⁻¹ with a lower limit of detection of 0.156–10 ng/mL [3, 4].

### Apoptosis analysis by flow cytometry

The apoptotic status of BMDMs was assessed using the Annexin V-FITC Apoptosis Detection Kit (BD Pharmingen). This involved dual staining of cells with Annexin V-FITC and PI to differentiate between apoptotic and necrotic cells. Subsequent analysis was conducted on a BD LSR II flow cytometer (BD Biosciences), with 20,000 cells enumerated per sample. Data analysis was performed using FlowJo software (BD Life Sciences) to quantify apoptotic rates across different experimental conditions.

### Macrophage subtype analysis by flow cytometry

For macrophage immunophenotyping, cells were gently detached using a cell scraper and resuspended at a concentration of 2×10^6^ cells/mL. The cells were then incubated with a combination of antibodies for 30 min at 4 °C. Subsequently, the cells were washed twice with PBS supplemented with 2% fetal bovine serum (FBS). M1 macrophages were characterized using F4/80-APC and CD86-FITC antibodies, while M2 macrophages were identified using F4/80-APC and CD206-PE-Cy5 antibodies (BD Biosciences). Data acquisition was performed on a BD LSR II flow cytometer, and data were analyzed using FlowJo software. Specific gating strategies are outlined in the figure legends.

### Protein expression analysis by western blot

Protein was extracted from snap-frozen pulmonary homogenates and whole-cell lysates, and quantified as previously described. Equal amounts of protein (30 μg per sample) were loaded onto SDS-PAGE gels and transferred to PVDF membranes. The membranes were then probed with primary antibodies specific to NLRP3(Cell Signaling Technology, catalog no.15101), GSDMD(Cell Signaling Technology, catalog no.39754), GSDMD-NT(Cell Signaling Technology, catalog no.39754), Caspase-1(Cell Signaling Technology, catalog no.83383), IL-1β(ZenBio, catalog no.516288), β-actin (Santa Cruz Biotechnology, catalog no.sc-517582), and β-tubulin (Proteintech, catalog no.10094-1-AP). Subsequently, secondary antibodies were applied, and immunoreactive bands were visualized using horseradish peroxidase. Band intensities were analyzed using a Kodak Scientific Imaging System, with β-actin/β-tubulin serving as an internal loading control.

### Cytokine quantification by ELISA

Cytokines and inflammatory mediators in culture supernatants and mouse serum were quantified using ELISA kits from R&D Systems targeting CXCL2, CCL3, IL-1β, IL-18, TNF-α, IL-6, and eCIRP. Samples were applied to pre-coated ELISA plates, incubated with specific antibodies, and a substrate was added to produce a colorimetric signal corresponding to the concentration of the target protein. Optical density was measured at 450 nm, and cytokine concentrations were determined using standard curve analysis.

### LDH release assay

LDH release was assessed with the Pierce™ LDH Cytotoxicity Assay Kit (Thermo Fisher Scientific). Supernatants from BMDM cultures were collected, centrifuged at 500 × *g* for 6 min, and absorbance at 490 nm was measured on a BioTek Synergy 2 plate reader. The cytotoxicity index was calculated as (LDH in medium/total LDH) × 100%.

### Assessment of plasma membrane integrity by PI staining

BMDMs cultured in 96-well plates were incubated with PI solution (1:300 dilution). PI fluorescence (membrane disruption) and bright-field images were acquired directly in the plate using an Incucyte live-cell imaging system; PI-positive cells were quantified with Incucyte software and expressed as the percentage of total cells.

### Immunofluorescence analysis

Paraffin-embedded sections from different treatment groups were first incubated with appropriate primary antibodies: anti-GSDMD (1:500, CST), followed by incubation with secondary antibodies. Subsequently, nuclear staining was performed using DAPI (Sigma). During the staining process, a negative control was prepared by omitting the GSDMD primary antibody. The sections were visualized using fluorescence confocal microscopy and subjected to quantitative analysis using WCIF ImageJ software (National Institutes of Health, Bethesda, MD). For each tissue section, 20 high-power fields were examined.

### Phagocytosis assessment

Macrophages’ phagocytic ability was assessed using FITC-labeled zymosan bioparticles. Prior to zymosan particle introduction, BMDMs were activated with LPS. Phagocytosis was quantified using flow cytometry (FACS) and reported as the phagocytosis index (PhI). This approach facilitated a prompt and accurate evaluation of macrophage phagocytic function, providing a quantitative assessment of their functional state across various experimental settings.

### Antimicrobial function of BMDMs

Fresh overnight cultures of Escherichia coli, Candida albicans and Cronobacter sakazakii were adjusted to OD_600_ = 0.2. BMDMs were pretreated with disulfiram at indicated concentrations for 12 h, after which bacteria were added at an MOI of 10 (corresponding to 1 × 10⁷ CFU per well). After overnight co-culture, CFUs were enumerated to assess bacterial proliferation. This protocol systematically examines macrophage–bacteria interactions in the presence of disulfiram, providing a robust platform for dissecting macrophage functional modulation and bacterial growth inhibition.

### Statistical analysis

The data were analyzed using GraphPad Prism software (version 4). Quantitative data were expressed as mean ± SD and subjected to thorough statistical analysis. Normality was checked with the Shapiro–Wilk test; and one-way ANOVA was used for normally distributed data (Shapiro–Wilk test, *P* > 0.05). Initial analyses comprised Student’s *t*-test or ANOVA, with Bonferroni’s multiple comparison test applied when appropriate. Survival data were assessed using the log-rank (Mantel–Cox) test. Post-hoc power for the log-rank test was 0.85 (*α* = 0.05) given the observed hazard ratio of 3.2 and 8 animals per group. Statistical significance was defined as *P* < 0.05.

## Supplementary information

Figure S1. GSDMD Knockout Alters Macrophage Phenotype and Activation (related to Figure 3)

Figure S2. GSDMD Knockout Inhibits Pyroptotic Stimuli-Induced IL-1β Release in Macrophages (related to Figure 5)

Figure S3. ROS Drive Macrophage Pyroptosis via GSDMD N-Terminal Cleavage and Pore Formation (related to Figure 7)

Table 1. The primer sequences for the real-time PCR measurement

The original images of the WB (Western Blot) experiments were not cropped and were integrated as a whole.

The original, uncropped Western blot images for Figures S2 and S3 are presented in their entirety.

## Data Availability

The data that support the findings of this study are available from the corresponding author upon reasonable request.
