# Supplementary material for: GSDMD deficiency attenuates BPD by suppressing macrophage pyroptosis and promoting M2 polarization
Source: Cell Death Discov. 2025 Dec 4;12:33. doi: 10.1038/s41420-025-02872-4 (PMC12824217; doi:10.1038/s41420-025-02872-4)
Supplement: Supplementary file 1 — Figure S1. GSDMD Knockout Alters Macrophage Phenotype and Activation (related to Figure 3) [file 41420_2025_2872_MOESM1_ESM.docx]

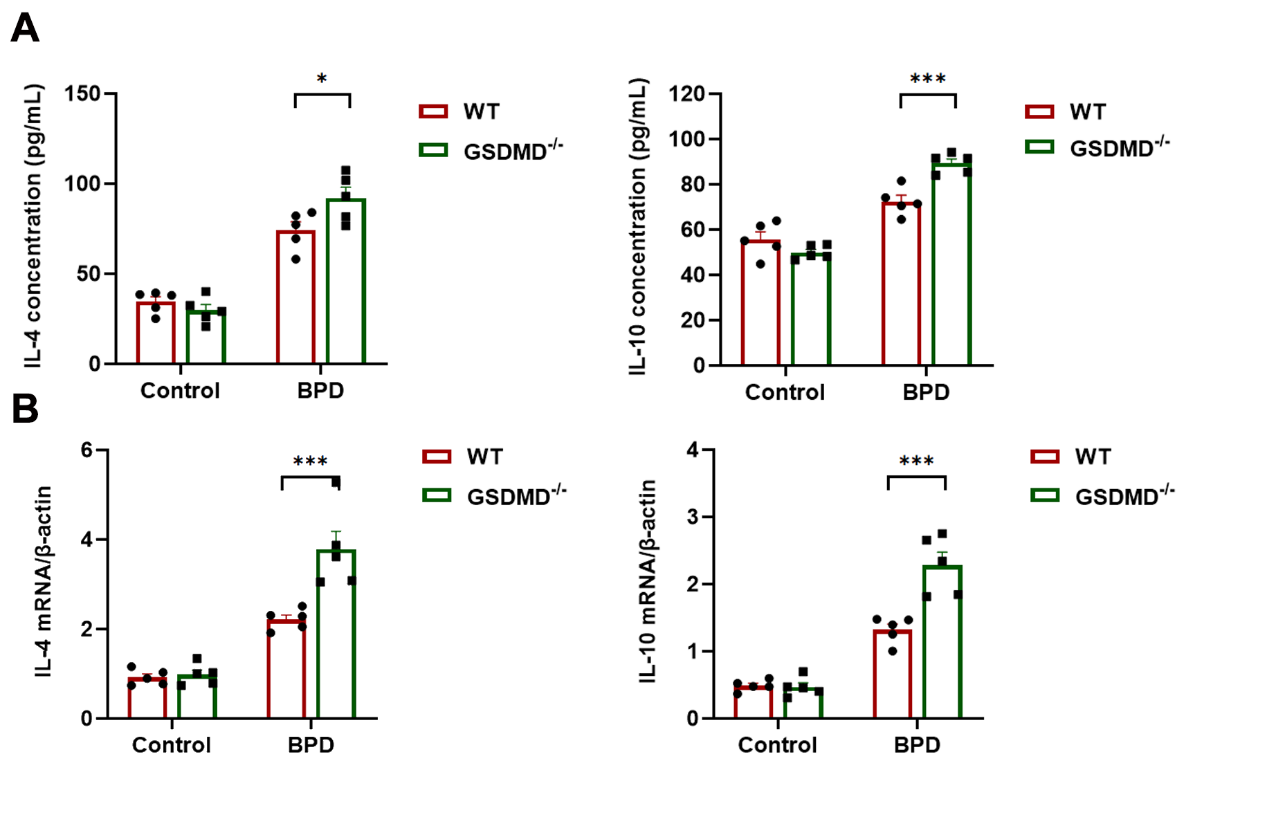


**Figure S1.** **GSDMD Knockout Alters Macrophage Phenotype and Activation（related to Figure 3）**

A. IL-4 and IL-10 levels in cell-free BALF were measured by ELISA. Columns depict the mean ± SD of multiple independent experiments (n = 5 mice per group); *P < 0.05, ***P < 0.001, one-way ANOVA. B. qRT-PCR was used to quantify IL-4 and IL-10 mRNA in lung tissue from each treatment group, with values normalized to β-actin mRNA (n = 5). Columns show the mean ± SD of multiple independent experiments; ***P < 0.001, one-way ANOVA.
