# Supplementary material for: GSDMD deficiency attenuates BPD by suppressing macrophage pyroptosis and promoting M2 polarization
Source: Cell Death Discov. 2025 Dec 4;12:33. doi: 10.1038/s41420-025-02872-4 (PMC12824217; doi:10.1038/s41420-025-02872-4)
Supplement: Supplementary file 2 — Figure S2. GSDMD Knockout Inhibits Pyroptotic Stimuli-Induced IL-1β Release in Macrophages (related to Figure 5) [file 41420_2025_2872_MOESM2_ESM.docx]

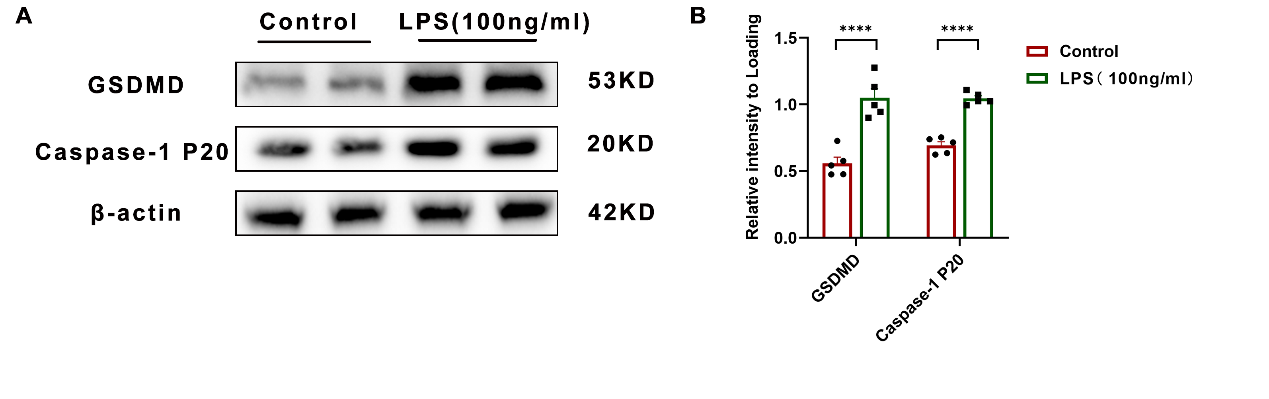


**Figure S2. GSDMD Knockout Inhibits Pyroptotic Stimuli-Induced IL-1β Release in Macrophages (****related to Figure 5)**

A. Western blot was performed to assess full-length GSDMD and the cleaved caspase-1 p20 fragment in bone-marrow-derived macrophages (BMDMs).Representative blots from three independent experiments with consistent results are shown. B. Bands were quantified using ImageJ. Data are presented as mean ± SD from three biologically independent samples; ****P < 0.0001, two-way ANOVA.
