# Supplementary material for: GSDMD deficiency attenuates BPD by suppressing macrophage pyroptosis and promoting M2 polarization
Source: Cell Death Discov. 2025 Dec 4;12:33. doi: 10.1038/s41420-025-02872-4 (PMC12824217; doi:10.1038/s41420-025-02872-4)
Supplement: Supplementary file 3 — Figure S3. ROS Drive Macrophage Pyroptosis via GSDMD N-Terminal Cleavage and Pore Formation (related to Figure 7) [file 41420_2025_2872_MOESM3_ESM.docx]

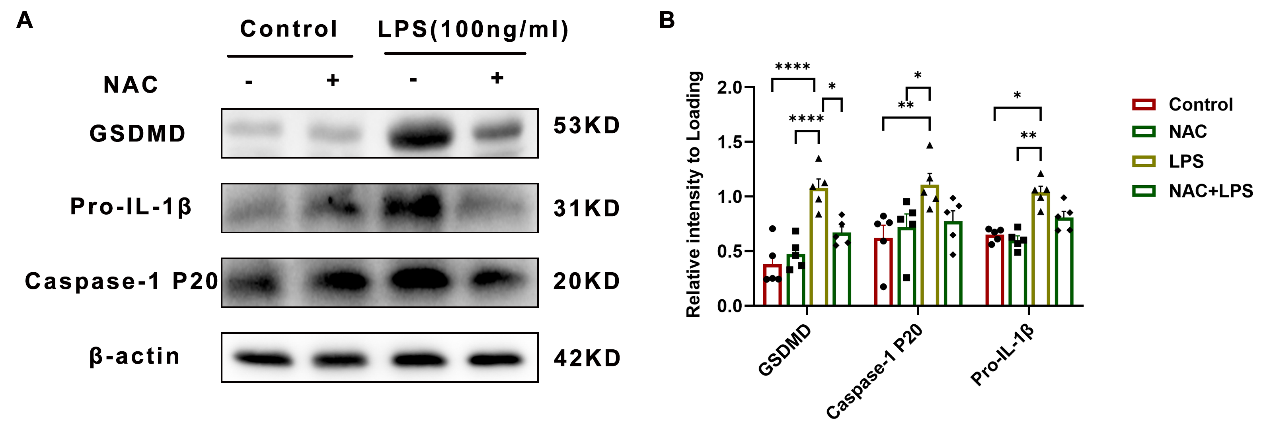


**Figure S3. ROS Drive Macrophage Pyroptosis via GSDMD N-Terminal Cleavage and Pore Formation (****related to Figure 7)**

A. Western blotting was used to assess the expression of full-length GSDMD, cleaved caspase-1 p20, and pro-IL-1β in bone-marrow-derived macrophages (BMDMs). Representative blots from three independent experiments with consistent results are shown. B. Bands were quantified using ImageJ. Data are presented as mean ± SD from three biologically independent samples; *P < 0.05, **P < 0.01, ****P < 0.0001, two-way ANOVA.
