# Supplementary material for: GSDMD deficiency attenuates BPD by suppressing macrophage pyroptosis and promoting M2 polarization
Source: Cell Death Discov. 2025 Dec 4;12:33. doi: 10.1038/s41420-025-02872-4 (PMC12824217; doi:10.1038/s41420-025-02872-4)
Supplement: Supplementary file 4 — Table 1. The primer sequences for the real-time PCR measurement [file 41420_2025_2872_MOESM4_ESM.pdf]

Table 1. The primer sequences for the real-time PCR measurement

Genes	Forward primer	Reverse primer
Mouse IL-1β	AGTGTGGATCCCAAGCAATACCCA	TGTCCTGACCACTGTTGTTTCCCA
Mouse IL-6	CCAATTTCCAATGCTCTCCT	ACCACAGTGAGGAATGTCCA
Mouse CD86	TGGAGAGGGAAGAGAGTGAACA	GCCCATAAGTGTGCTCTGAA
Mouse iNOS	GTTCTCAAGGCACAGGTCTC	GCAGGTCACTTATGTCACTTATC
Mouse CD206	CCATGGACAATGCGCGAGCG	CACCTGTGGCCCAAGACACGT
Mouse Arg-1	TTCTCAAAAGGACAGCCTCG	GCTCTTCATTGGCTTTCCC
Mouse TNF-α	ATGGCCTCCCTCTCATCAGTT	ACAGGCTTGTCACTCGAATTTTG
Mouse IL-10	CAGGCAGAGAAGCATGGC	TGCTCCACTGCCTTGCTC
Mouse β-actin	CCCTGGAGAAGAGCTACGAG	CGTACAGGTCTTTGCGGATG
