# Supplementary material for: GSDMD deficiency attenuates BPD by suppressing macrophage pyroptosis and promoting M2 polarization
Source: Cell Death Discov. 2025 Dec 4;12:33. doi: 10.1038/s41420-025-02872-4 (PMC12824217; doi:10.1038/s41420-025-02872-4)

Full Blot of Figure.5A

GSDMD-NT

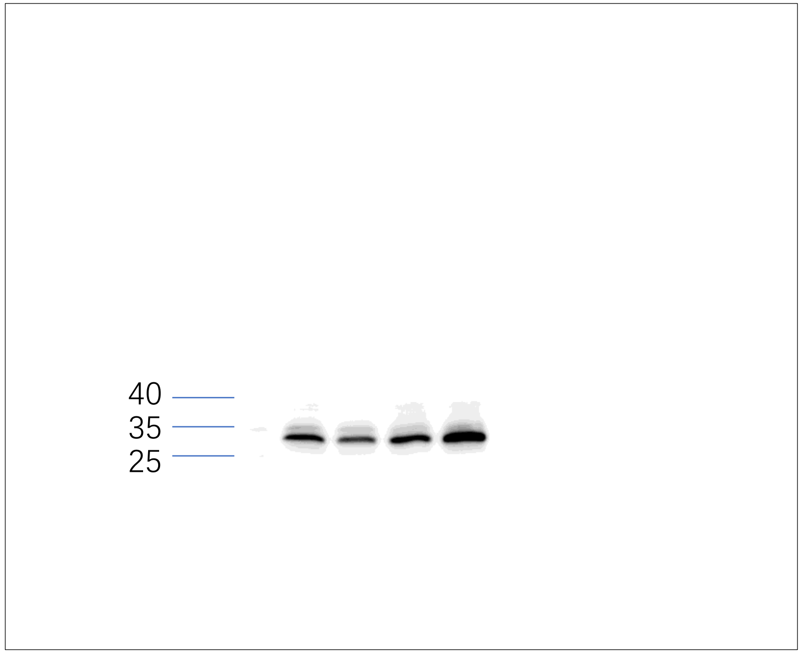

β-actin

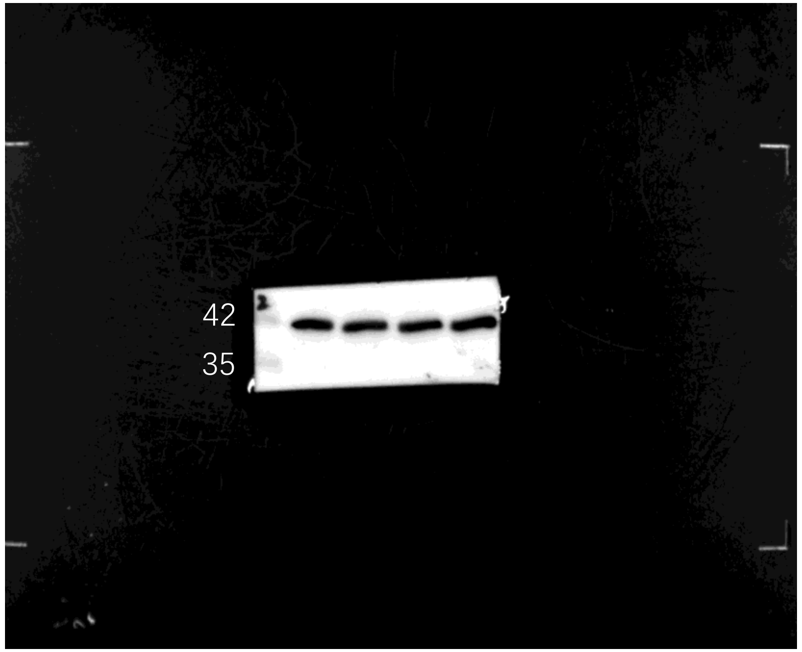

Caspase-1

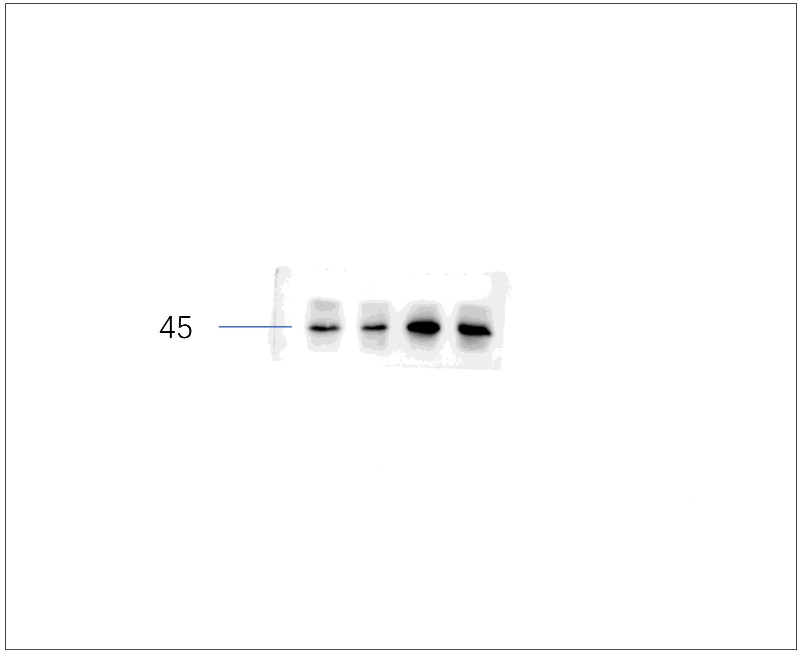

β-actin

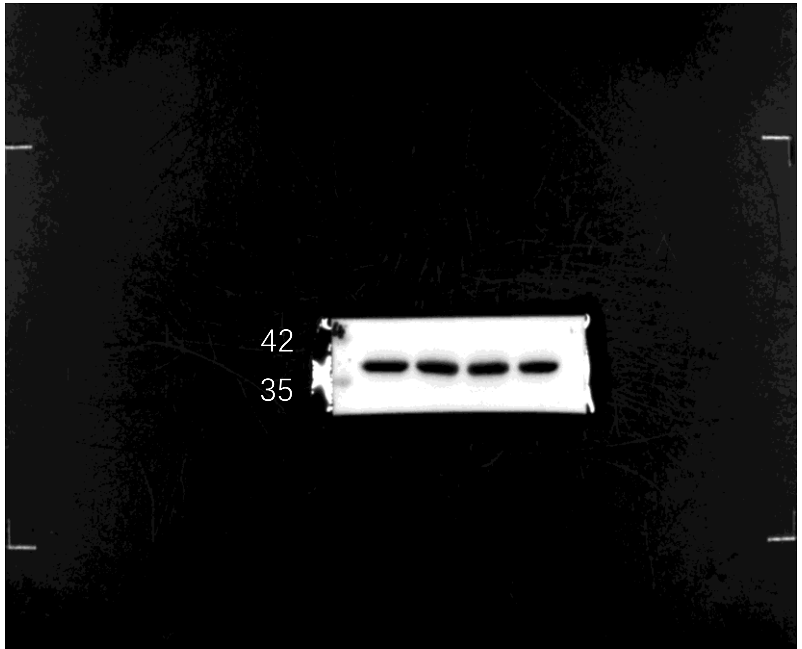

Full Blot of Figure.7D

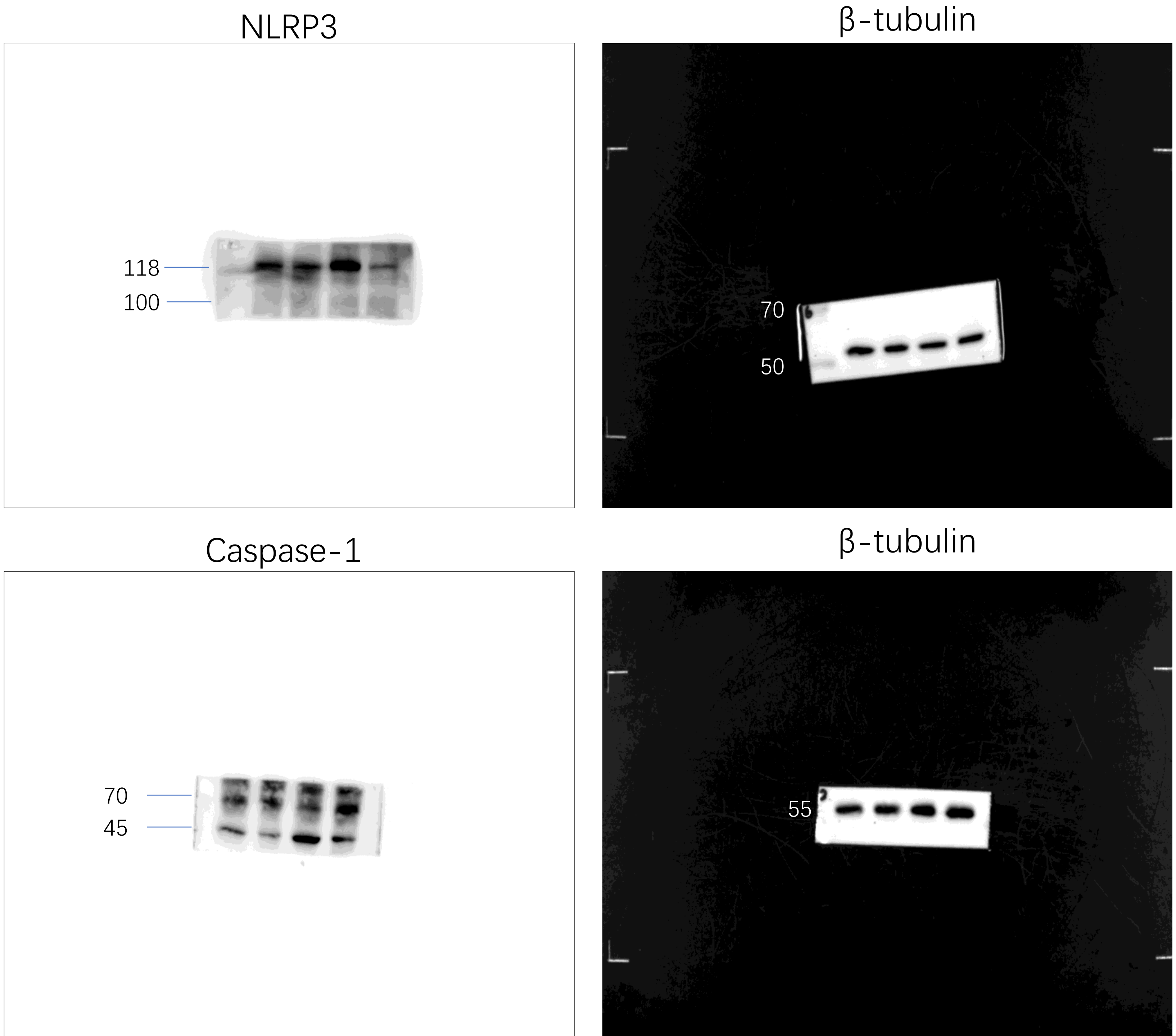

GSDMD-NT

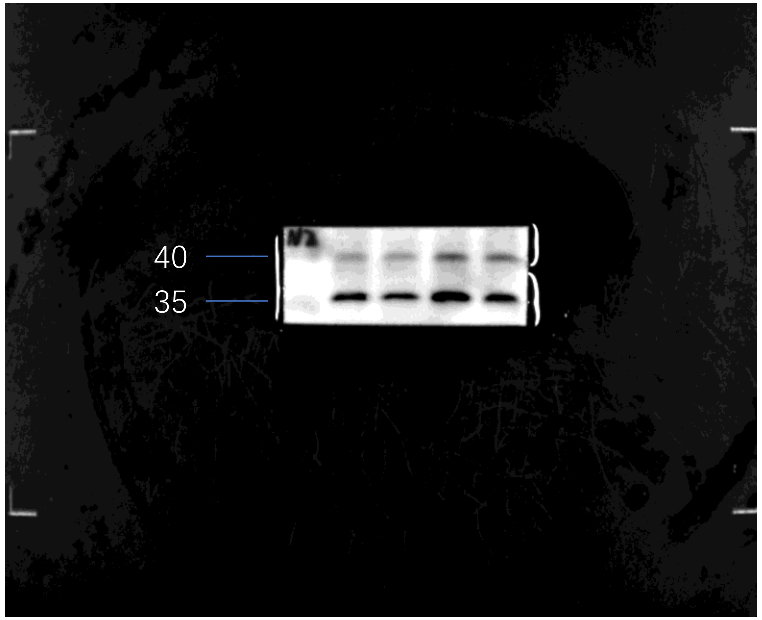

β-tubulin

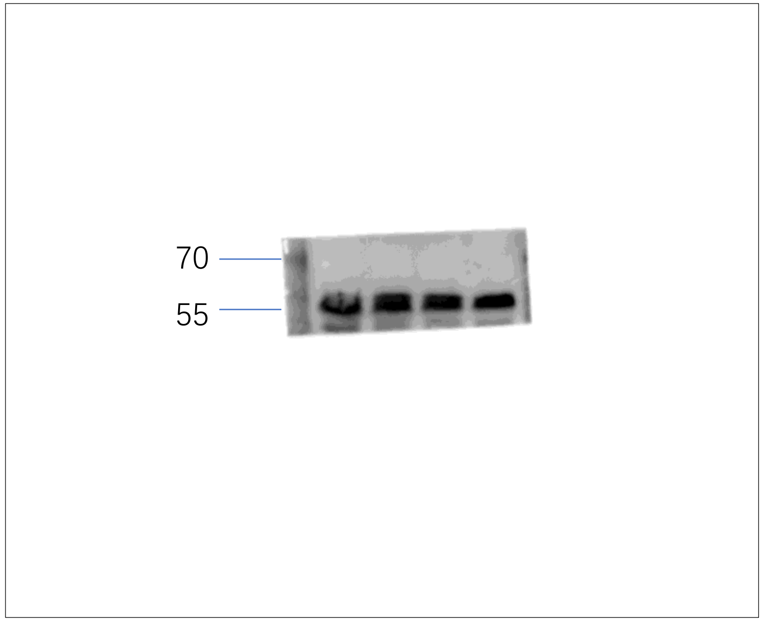

IL-β

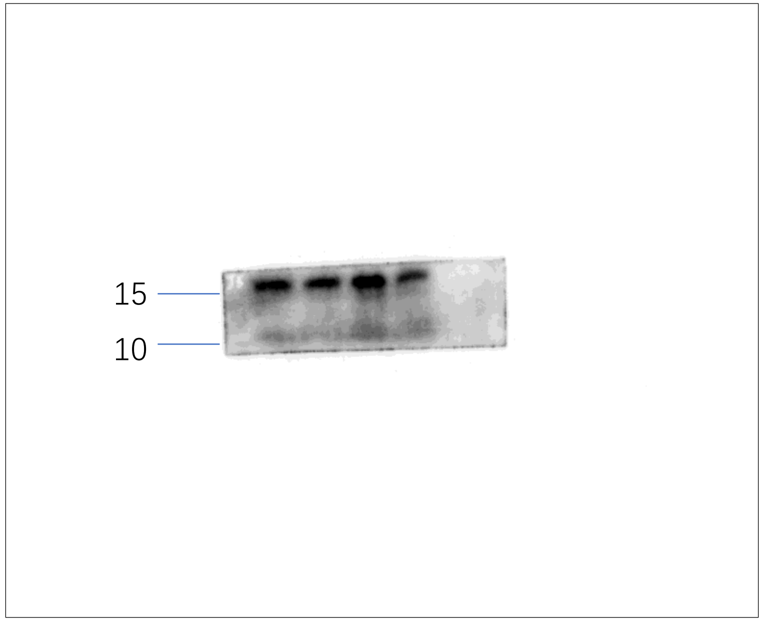

β-tubulin

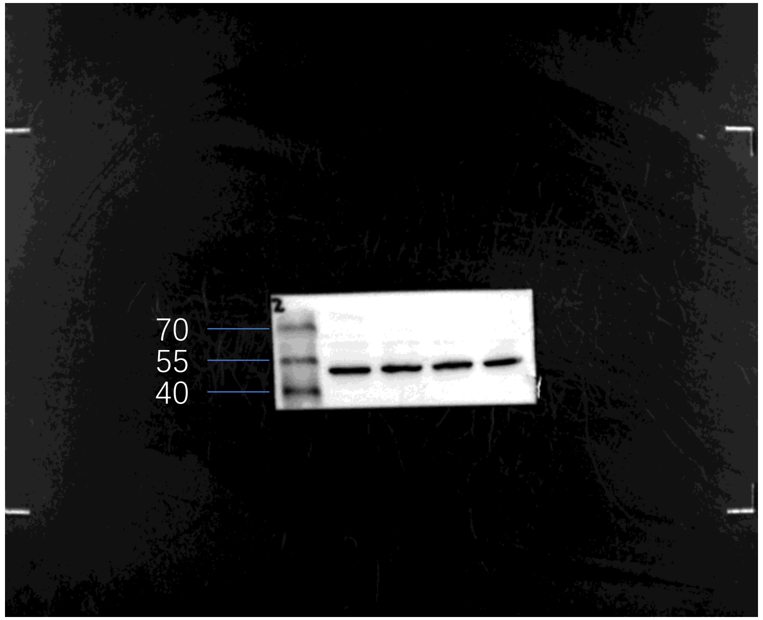

Supplement: Supplementary file 5 — The original images of the WB (Western Blot) experiments were not cropped and were integrated as a whole. [file 41420_2025_2872_MOESM5_ESM.pdf]
