# Supplementary material for: GSDMD deficiency attenuates BPD by suppressing macrophage pyroptosis and promoting M2 polarization
Source: Cell Death Discov. 2025 Dec 4;12:33. doi: 10.1038/s41420-025-02872-4 (PMC12824217; doi:10.1038/s41420-025-02872-4)

Full Blot of Figure.S2

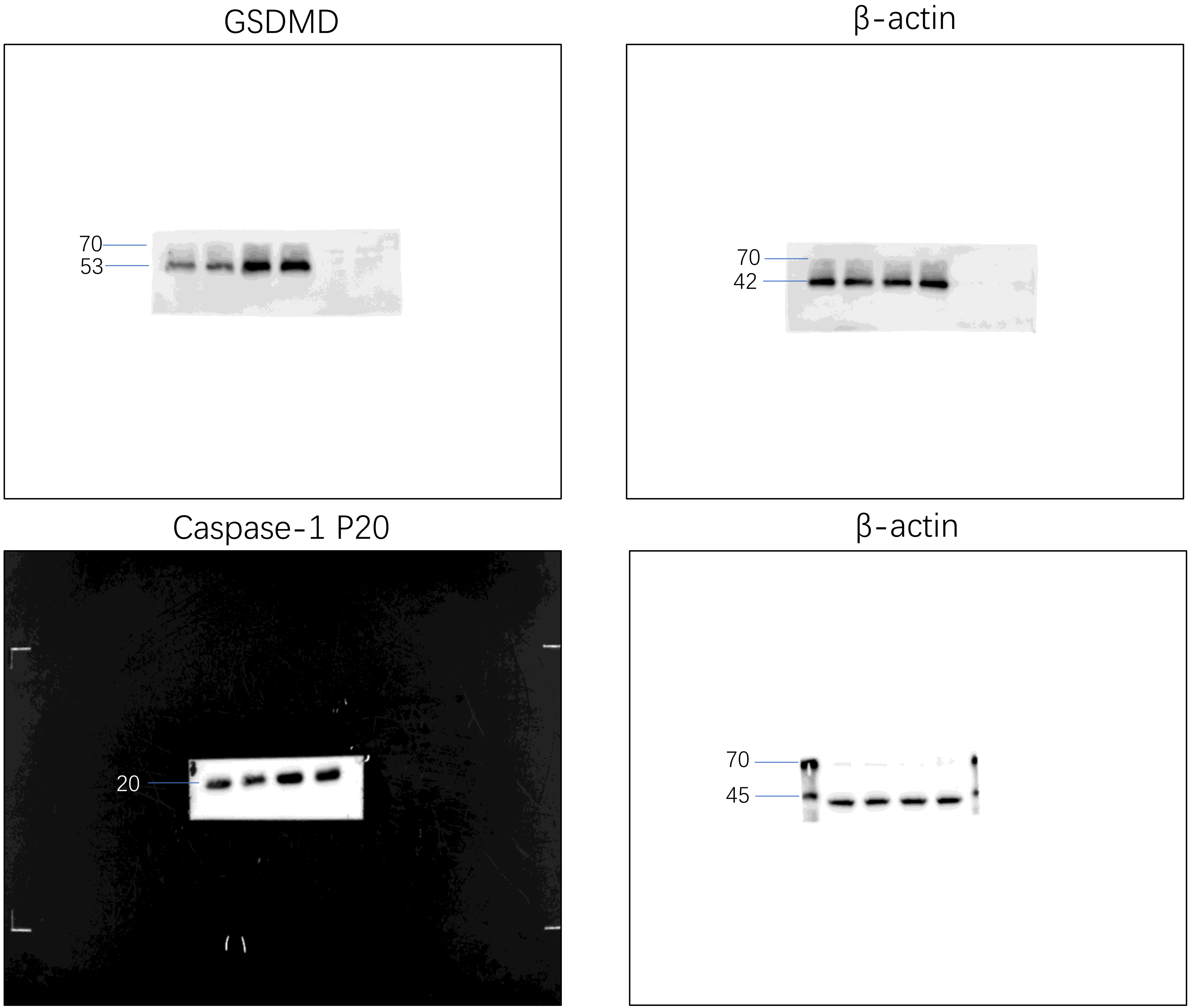

Full Blot of Figure.S3

GSDMD

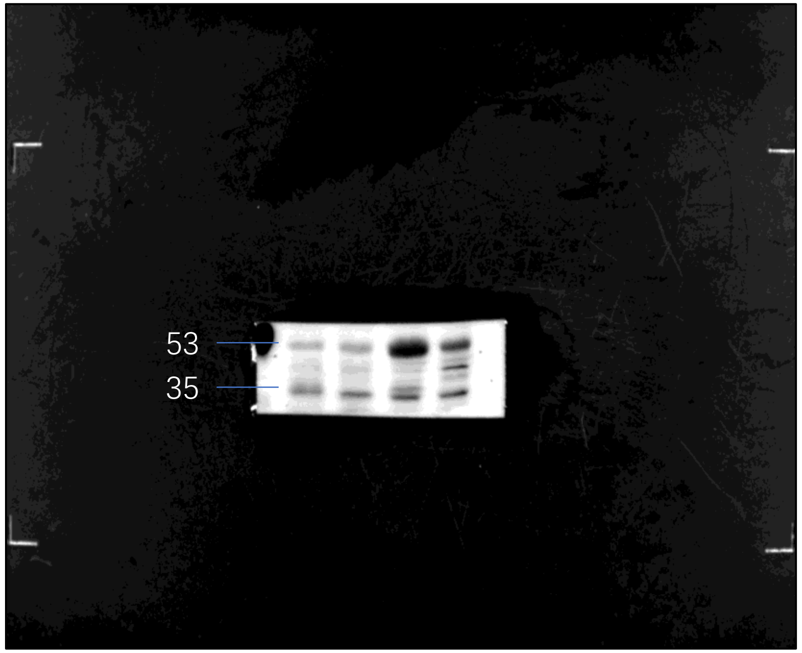

β-actin

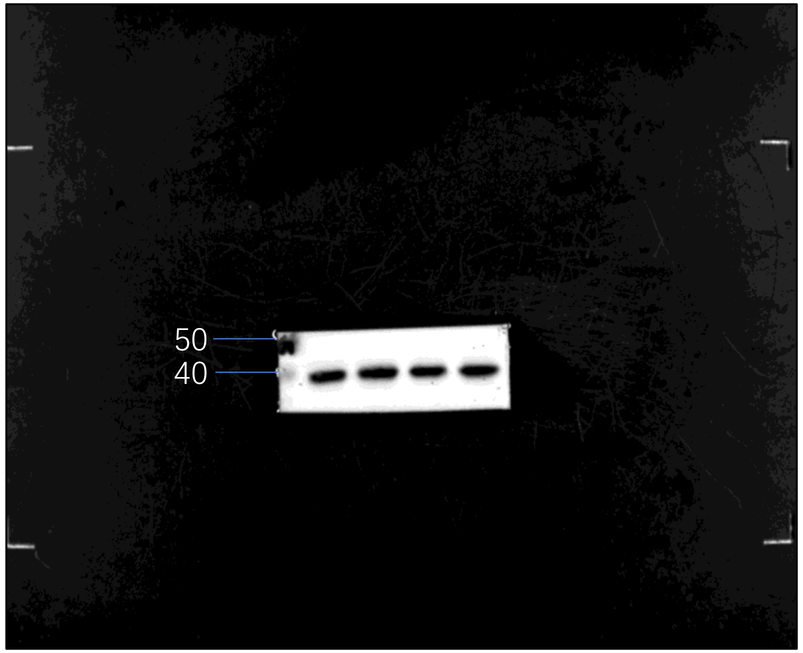

Pro-IL-1β

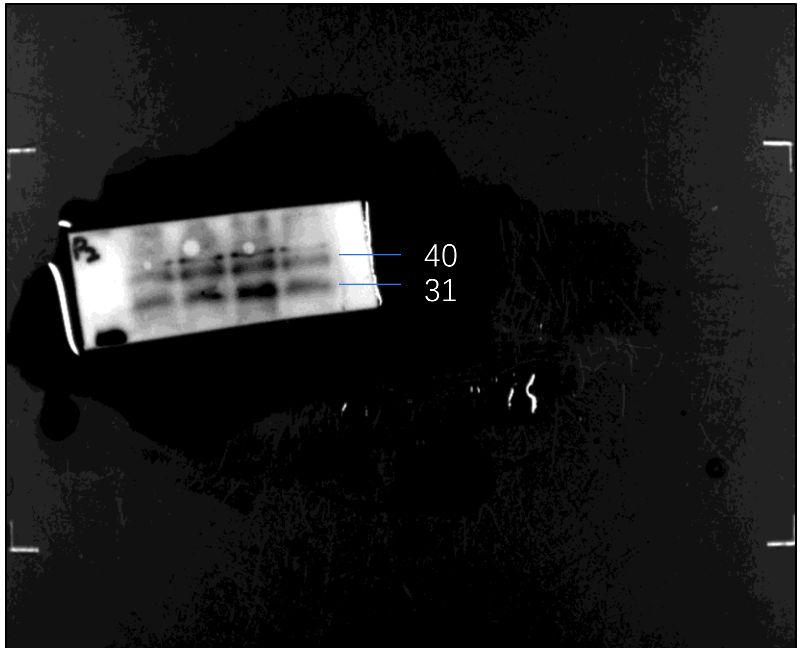

β-actin

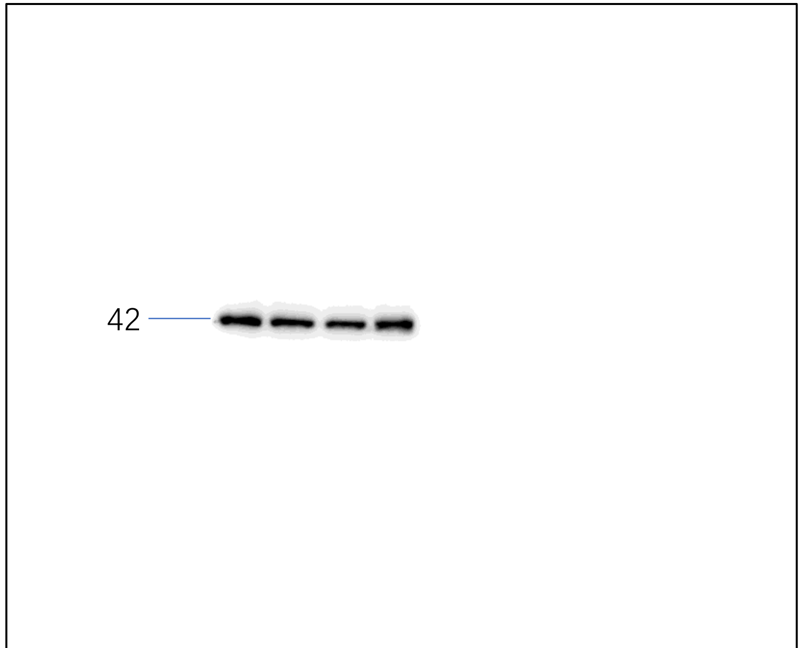

Caspase-1 P20

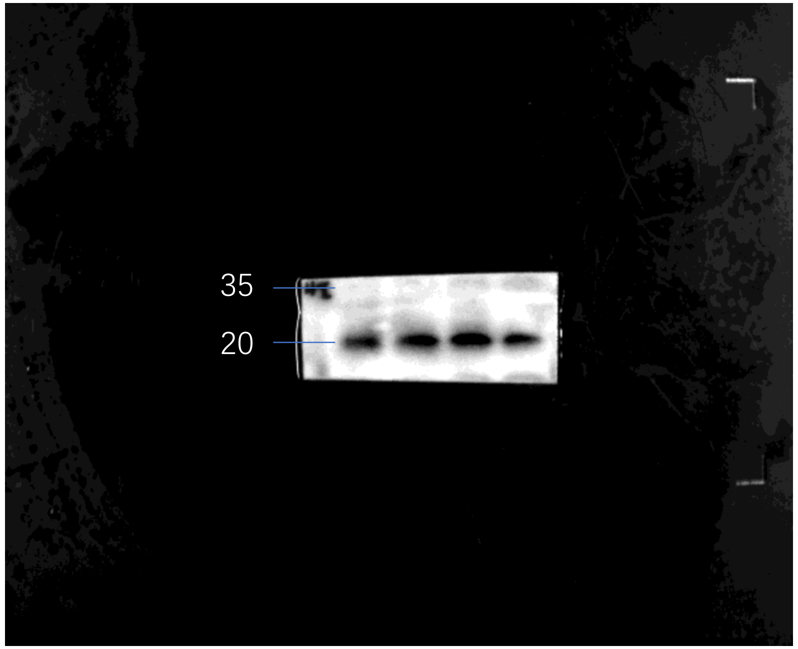

β-actin

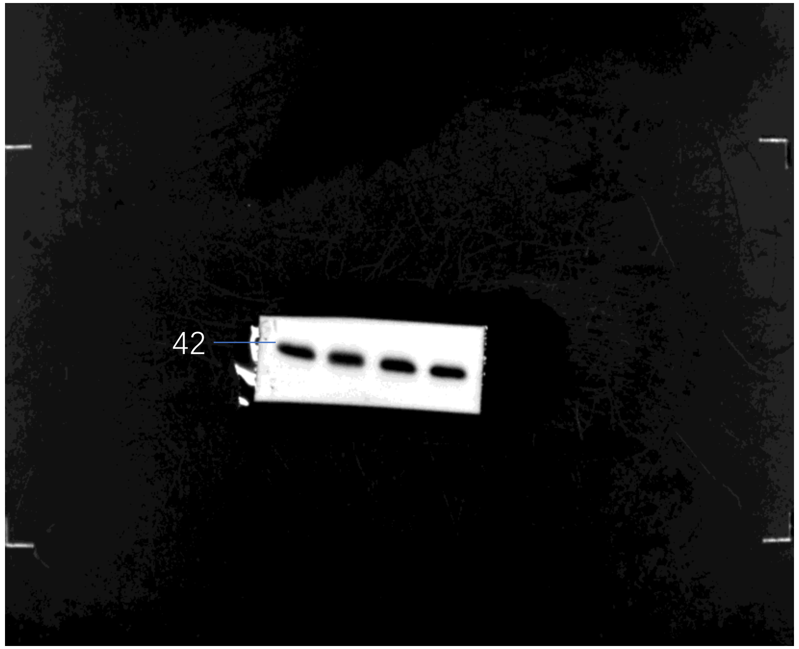

Supplement: Supplementary file 6 — The original, uncropped Western blot images for Figures S2 and S3 are presented in their entirety. [file 41420_2025_2872_MOESM6_ESM.pdf]
